# Deciphering the antimicrobial activity of multifaceted rhizospheric biocontrol agents of solanaceous crops *viz.*, *Trichoderma harzianum* MC2, and *Trichoderma harzianum* NBG

**DOI:** 10.3389/fpls.2023.1141506

**Published:** 2023-03-03

**Authors:** Mehjebin Rahman, Sapna Mayuri Borah, Pradip Kr. Borah, Popy Bora, Bidyut Kumar Sarmah, Milan Kumar Lal, Rahul Kumar Tiwari, Ravinder Kumar

**Affiliations:** ^1^Department of Plant Pathology, Assam Agricultural University, Jorhat, Assam, India; ^2^Department of Plant Pathology, Regional Agricultural Research Station, Jorhat, Assam, India; ^3^Department of Agricultural Biotechnology, Assam Agricultural University, Jorhat, India; ^4^Department of Plant Protection; Department of Crop Physiology, Biochemistry & Postharvest Technology, ICAR-Central Potato Research Institute, Shimla, India

**Keywords:** solanum, morpho-molecular pathology, LC-MS, electron microscopy, soil-borne antagonists

## Abstract

The *Solanaceae* family is generally known to be the third most economically important plant taxon, but also harbors a host of plant pathogens. Diseases like wilt and fruit rot of solanaceous crops cause huge yield losses in the field as well as in storage. In the present study, eight isolates of *Trichoderma* spp. were obtained from rhizospheric micro-flora of three solanaceous crops: tomato, brinjal, and chili plants, and were subsequently screened for pre-eminent biocontrol activity against three fungal (*Fusarium oxysporum* f. sp. *lycopersicum*, *Colletotrichum gloeosporioides*, and *Rhizoctonia solani*) and one bacterial (*Ralstonia solanacearum*) pathogen. Morphological, ITS, and *tef1α* marker-based molecular identification revealed eight isolates were different strains of *Trichoderma*. Seven isolates were distinguished as *T. harzianum* while one was identified as *T. asperellum*. *In vitro* antagonistic and biochemical assays indicated significant biocontrol activity governed by all eight isolates. Two fungal isolates, *T. harzianum* MC2 and *T. harzianum* NBG were further evaluated to decipher their best biological control activity. Preliminary insights into the secondary metabolic profile of both isolates were retrieved by liquid chromatography-mass spectrometry (LC-MS). Further, a field experiment was conducted with the isolates *T. harzianum* MC2 and *T. harzianum* NBG which successfully resulted in suppression of bacterial wilt disease in tomato. Which possibly confer biocontrol properties to the identified isolates. The efficacy of these two strains in suppressing bacterial wilt and promoting plant growth in the tomato crop was also tested in the field. The disease incidence was significantly reduced by 47.50% and yield incremented by 54.49% in plants treated in combination with both the bioagents. The results of scanning electron microscopy were also in consensus with the *in planta* results. The results altogether prove that *T. harzianum* MC2 and *T. harzianum* NBG are promising microbes for their prospective use in agricultural biopesticide formulations.

## 1 Introduction

Agriculture in the 21^st^ century is encountering multifaceted challenges in two major sectors *viz.*, producing more food with a small labor rural force, and adapting sustainable production techniques to acclimatize to climate change (Agrimonti et al., 2021). Furthermore, the ever-increasing world population also necessitates enhanced food production. To meet the food security demands of a nation, the augmented requirements have demanded the development of innovative research and production technologies (Altaf et al., 2022; Lal et al., 2022a; Lal et al., 2022b; Su et al., 2023). One of the prime causes that barricade food grain production is biotic stress in plants. Most prevalent biotic stresses including bacterial, fungal, viral, nematode, and insect attacks in crops have attracted massive chemical-based pesticide consumption to avoid losses caused by them (Kumar et al., 2021; Mangal et al., 2022). Inadequate pesticide application harms the environment and has a negative impact on both beneficial and non-target soil microbiota (Bhardwaj et al., 2019). The continuous and rigorous use of chemicals also exerts high selection pressure on pathogens, resulting in mutations and the development of pesticide-resistant strains (Kumar et al., 2008a; Kumar et al., 2008b). In such a case, it is critical to employ alternative biological control strategies that have a lower ecological impact while maintaining yield. Such mechanisms are carried out by several beneficial and symbiotic associations between the plant and the microbes (Bonfante and Genre, 2010; Oldroyd, 2013; Kumar et al., 2014). One such class of fungal organisms that are widely considered for their application as biocontrol agents belong to the genus *Trichoderma* (Samuels, 1996; Sood et al., 2020).

*Trichoderma* spp. are predominant, opportunistic saprophytes, and ubiquitous constituents of the soil myco-biota found across all climatic zones. They are active and non-virulent components identified as plant symbionts (Harman et al., 2004). They are robust endophytes and are capable of colonizing roots and modulating the plant-soil-environment interactions either directly or indirectly (Martínez-Medina et al., 2013; Ghorbanpour et al., 2018; Tyśkiewicz et al., 2022). Additionally, they are well-known mycoparasites of numerous plant pathogens, thus enhancing plant defense and rendering resistance against the same (Hermosa et al., 2013). Additionally, *Trichoderma* spp. impart abiotic stress tolerance and promotes plant root growth by enhancing nutrient uptake and bioremediation of toxic heavy metals to avoid environmental pollution (Harman et al., 2008). *Trichoderma* are also known to release secondary metabolites and enzymes with industrial application (Reino et al., 2008; Blaszczyk et al., 2014). Although this fungus has not yet produced any ‘wonder drug’ such as penicillin, these key factors together have driven plant pathologists and researchers worldwide towards exploiting *Trichoderma* as a commercial and state of art constituent of biopesticides. According to market figures, 60 percent of the successful and registered bio-fungicide production in India includes *Trichoderma* as an active bioagent (Kumar and Khurana, 2021).

*Trichoderma* are filamentous ascomycetes fungi that provide plants with several modes of antagonism to deter phyto-pathogenic growth (Kishan et al., 2017). A prominent mechanism of antagonism is the release of compounds like polysaccharide-degrading enzymes that induce systemic acquired resistance (SAR) and induced systematic resistance (ISR) in plants (Pal and Gardener, 2006; Stirling, 2018). Coiling of pathogenic hyphae by the fast-growing hyphae of *Trichoderma* promotes mycoparasitism (Inbar et al., 1996; Almeida et al., 2007). Several enzymes secreted by *Trichoderma* assist mycoparasitism such as chitinase and protease (Geremia et al., 1993; Carsolio et al., 1999). Additionally, *Trichoderma* also release antibiotics like trichodermin, suzukacillin, and alamethicin, which stimulate physiological and morphological changes leading to successful penetration of hyphae (Dennis and Webster, 1971a; Ghisalberti and Sivasithamparam, 1991; Krause et al., 2006; Dotson et al., 2018). An active form of antagonism is exhibited by competition in the rhizosphere for food and space (Nakkeeran et al., 2018). For instance, *Trichoderma* initiate siderophore release that limits the availability of iron to pathogens by chelating Fe^2+^ ions forming siderophore-Fe complex recognized primarily by the membrane-bound protein receptors of biocontrol agents alone (Anke et al., 1991; Pandey et al., 2010; Vinale et al., 2013). Specific metabolic pathways like phytoalexin biosynthesis and phenylpropanoid metabolism accelerate a hypersensitive response in *Trichoderma* (Contreras-Cornejo et al., 2014; Tripathi et al., 2021). With the advent of molecular biology techniques, new strains of *Trichoderma* have been identified based on ribosomal DNA Internal Transcribed Spacer (ITS) region (ITS1—5.8S rDNA—ITS2), and fragments of genes encoding for the translation elongation factor 1-alpha (*tef-1α*), endochitinase (chi18-5), RNA polymerase II subunit (rpb2), and calmodulin (cal1) (Gupta et al., 2014).

The current work includes the successful isolation of rhizospheric *Trichoderma* strains from various North-eastern regions of Assam, India. The comprehensive workflow includes morphological, molecular, and biochemical characterization; analysis of the *in vitro* inhibitory effect of the strains against major bacterial and fungal pathogens of solanaceous crops; and *in planta* experiments and evaluation of the plant growth-promoting characteristics. To further corroborate antimicrobial activity, liquid chromatography-mass spectroscopy (LC137 MS) was pipelined accordingly. For in-depth studies of successful *in silico* experiments, scanning electron microscopy was carried out to visualize *Trichoderma* colonization in healthy roots. The experimental and in silico findings in the present work provide a holistic approach to assess *Trichoderma* isolates as potential bio-control agents for the future development of pesticide bio-formulations.

## 2 Materials and methods

### 2.1 Isolation and selection of rhizospheric biocontrol fungus

Soil samples with minimal use of pesticides were used in this study. Samples were collected from several vegetable sites in Assam across eight target districts, namely, Sibsagar, Jorhat, Tinsukia, Darrang, Sonitpur, Kamrup, Nalbari, and Barpeta. Rhizospheric soil (10g), individually sampled from disease-free crops of tomato, brinjal, and chili, was collected from all the locations at depths of 5 to 15cm followed by the removal of debris. Microbes were isolated by the standard serial dilution plate technique of Johnson and Curl, from 1g of tightly adhering rhizospheric soil (Johnson and Curl, 1972). Isolated rhizospheric fungi were purified and preserved in PDA slants at 4°C by subculturing at an interval of 1 month. All fungal isolates were screened to assess antagonistic properties against three fungal pathogens *F. oxysporium* f.sp. *lycopersicum* (NAIMCC-F-02784)*, Colletotrichum gloeosporioides* (accession OM202512), and *Rhizoctonia solani* JB1, and one bacterial pathogen *R. solanacearum* NP3. These pathogens were morphologically identified based on standard references, and the pathogenicity assay was also performed on host plants (Leslie and Summerell, 2006; Fiers et al., 2012). *In vitro* tests to estimate the inhibitory effect was conducted using PDA as basal media for fungal pathogens using dual culture assay. The mycelial bits (5 mm diameter) of bioagent and pathogens were excised from respective pure cultures and kept on individual plates to observe the inhibition percentage. Additionally, triphenyl tetrazolium chloride (TTC) medium was utilized for *R. solanacearum* for inhibitory assay technique (Aspiras and Cruz, 1985). A three-day-old culture of *R. solanacearum* suspension maintained with a concentration of 2 × 10^8^ CFU/ml was seeded on TTC media and subsequently incubated with mycelial bits of bioagents. The resultant percentage of growth inhibition was calculated (Erdogan and Benlioglu, 2010). The experiment was conducted in two replicates.

### 2.2 Characterization and identification of potent rhizospheric fungi

Promising fungal microbes showing predominant activity against the targeted pathogens of solanaceous crops retrieved from the above analysis were subjected to morph-cultural characterization. All the isolates were grown on PDA media and incubated in darkness for 3 days at 28 ± 2C. The morphology of conidiophores, shape and size of phialides, and conidial characteristics were studied using a Leica light compound microscope at 10X and 40X magnification. The cultural characteristics, such as growth, colony color, texture, pigmentation, ring formation, and margin orientation were also documented. Microbial DNA was isolated by CTAB-phenol–chloroform-isoamyl alcohol method which was further used as the template DNA for Polymerase Chain Reaction (PCR). Approximately 650bp of the *Trichoderma* ITS region was amplified using universal primers ITS1 (5′-GGAAGTAAAAGTCGTAACAAGG-3′) and ITS4 (5′ TCCTCCGCTTATTGATATGC 3′) (White et al., 1990). Amplifications were carried out in a PCR thermal cycler (Thermo Fisher Scientific) using an initial denaturation at 94°C for 1 min followed by 30 cycles of denaturation for 35 sec at 94°C, annealing for 1 min at 57°C and extension for 1 min at 72°C. This was concluded with a final extension for 10 min at 72°C. A similar attempt was made to amplify the *tef1α* gene using the forward primer EF1 (5´- ATGGGTAAGGAGGACAAGAC3) and reverse primer (GCCATCCTTGGAGATACCAGC) (Samuels et al., 2002) with an initial denaturation at 95°C for 3 min followed by 30 cycles of denaturation for 1 min at 95°C, annealing for 1 min at 60°C, extension for 1 min at 72°C and final extension for 10 min at 72°C. The PCR products were confirmed on 1% agarose gel and purified using a DNA Gel Extraction Kit (Qiagen) prior to DNA sequencing (Eurofins Genomics India Pvt. Ltd., Bengaluru, Karnataka). The sequences were identified from GenBank database (Benson et al., 2012) of National Centre for Biotechnology Information (NCBI) (Wheeler et al., 2007) using a basic local alignment search tool (Johnson et al., 2008).

### 2.3 Study of antimicrobial properties of the potent rhizospheric fungi

#### 2.3.1 Antibiosis test for the production of volatile inhibitory compounds

Fungal strains were selected on the basis of their higher inhibition percentages against all four pathogens for further studies. The effect of volatile metabolites against the target pathogens was assessed according to the method of Dennis and Webster (Dennis and Webster, 1971b). Petri plates containing PDA medium were centrally inoculated with a 5 mm diameter disc from actively growing mycelia of antagonistic isolates. Another plate of the same diameter was inoculated with actively growing mycelia discs of test pathogens (both bacteria and fungus) and inverted over the first plate containing the antagonist disc. The junction of both the Petri dishes was tightly sealed and incubated at room temperature in such a way that the antagonists lay under the lower disc. Sealed dishes with the pathogen inoculated in one plate and no antagonist inoculated in the other plate of the pair were used as a control. The experiment was performed in triplicate replication. The percent inhibition was calculated by the formula given below:


$$Inhibition\;(\%)=\;\frac{\left(D1\;–\;D2\right)}{D1\;}\;x100$$

Where D1 stands for the fungus colony diameter growing in PDA (control), and D2 denotes fungus colony diameter growing in dual culture. For bacterial pathogens, colony-forming units were calculated and compared with the control.

#### 2.3.2 Antibiosis test

Isolates (*T. harzianum* MC2 and *T. harzianum* NBG) were inoculated in PDB in 100ml Erlenmayer flasks and incubated for 15 days at 25°C under agitation at 100 rpm on an orbital incubator. The culture broth was filtered through sterile filter paper to remove the mycelial mats and consecutively sterilized by passing through a 0.22μm pore biological membrane filter. The filtrate was blended with molten PDA medium to obtain 10, 25, and 50% (v/v) concentrations. A mycelial disc of 5mm diameter of each pathogen was put in the center of the Petri plates containing the blend and incubated at 25°C for 4-5 days. The plates without filtrate served as a control. The colony diameter was measured, and the percentage inhibition of the radial growth was calculated. Each assay was performed in triplicate.

#### 2.3.3 Qualitative assay of enzymes secreted by the rhizospheric microbes

An enzyme assay of the selected isolates (*T. harzianum* MC2 and *T. harzianum* NBG) was performed by plate assay on the corresponding solid media for the presence of extracellular enzymes. The bioagents were confirmed for the enzyme activity with the formation of clear zones, change of color, and intensity around the fungal colonies. Independent experiments with three replicates for each isolate were performed for the production of cellulase, amylase, and protease enzymes.

##### 2.3.3.1 Cellulase assay

Cellulase assay was done in triplicates for the antagonistic microbes (*T. harzianum* MC2 and *T. harzianum* NBG) by the method given by Hankin and Anagnostakis (Hankin and Anagnostakis, 1975). Mycelial discs of 5mm diameter of the 5-day-old cultures were transferred and incubated on the Czapek-Mineral Salt Agar Medium at 26 ± 2°C in darkness for 3 to 5 days. Upon achieving the required growth, the plates were then flooded with aqueous Congo red (2% w/v) solution for 15 min. The agar surface was then washed with distilled water and the plates were flooded with NaCl (1 M) for 1.5 min. Production of cellulase was confirmed by the formation of a yellow-opaque area around the colonies. The diameters of the colony and the clear zone were measured.

##### 2.3.3.2 Amylase assay

Sterilized medium of Starch Agar Medium (Hankin and Anagnostakis, 1975) was aseptically transferred to 90mm Petri dishes and inoculated with a 5mm agar disc cut from the 5-day-old fungal culture of each isolate separately and incubated at 26 ± 2°C in darkness for 3 to 5 days. Next, the plates were flooded with 1% iodine in 2% potassium iodide. The clear zone formed surrounding the colony was considered positive for amylase activity.

##### 2.3.3.4 Proteolytic assay

Proteolytic plate assay for the antagonistic microbes (*T. harzianum* MC2 and *T. harzianum* NBG) were done on Skimmed Agar media (Berg et al., 2005). The inoculation was done in a similar way to cellulose and amylase assays and incubated at 26 ± 2°C in darkness for 3 to 5 days. A clear halo formed around the colony showing positive proteolytic activity.

#### 2.3.4 Characterization of antimicrobial compound profile through LCMS

Liquid chromatography and mass spectroscopy were performed to analyze the secondary metabolite profile of the two best fungal bioagents in terms of qualitative assessment and inhibitory activity, *T. harzianum* MC2 and *T. harzianum* NBG. Sample preparation was done using a solvent extraction method. Fungi were individually inoculated in 250ml Erlenmeyer flasks containing 100ml of the medium and incubated at 28°C for one week with periodical shaking at 150rpm. After seven days of inoculation, the culture media were filtered in a solvent containing methanol: chloroform in the ratio of 1:2 and kept at 28 ± 1°C overnight. The lower aqueous layer was discarded, and the upper solvent layer was retained and concentrated in a rotary evaporator (IKA^®^, Staufen, Germany) to retrieve the crude compound. The dried powders were further dissolved in 2ml of HPLC-grade methanol and filtered through a 0.22µm syringe filter. LC-MS was done using Waters Alliance e2695/HPLC-TQD Mass spectrometer. 2µl injection volume was used and the compounds were separated on ACCUCORE C18 (150X4.6, 2.6µm, ACQ-TQD-QBB1152). Elution was done using 5% acetonitrile (solvent B), and 95% ammonium acetate (5 mM), mixed by Waters Alliance 2695 HPLC Pump. The flow rate was maintained at 0.6 ml/min. The total analysis time was 40 minutes. The column thermostat was maintained at 35°C while the autosampler equipment was operated at 20 °C. The eluted compounds were ionized using electrospray ionization (ESI) in the positive mode and detected using the MS detector coupled to the HPLC system. Data were scanned in the mass range of 200–2000m/z. The evaluation of the MS profile was performed using molecules downloaded from PubChem and analyzed *via* Mestrenova (MNOVA) software version 14.3.0 (Willcott, 2009).

### 2.4 *In planta* experiment

Assessment of plant growth promotion and disease suppression by two *T. harzianum* MC2 and *T. harzianum* NBG was done in Tomato variety Pusa ruby at Orchard located in Assam Agricultural University, Jorhat. Disease suppression was observed in artificially inoculated bacterial wilt caused by the pathogen *R. solanacearum*. Pathogen suspension was prepared in TTC broth and placed in a shaking incubator for 1 week at 25 ± 2°C, maintaining 10^8^ CFU ml^−1^. Pure cultures of *T. harzianum* MC2 and *T. harzianum* NBG were refreshed on PDA media for 4 days at 25°C. The inoculum was prepared in potato dextrose broth and placed in a shaking incubator for 1 week at 25 ± 2°C (Mwangi et al., 2011). Rhizospheric soil was dug up to 5 cm, and 5 ml of conidial suspension of 10^7^ ml^−1^ of *Trichoderma* was poured into the holes dug. Eight treatments (T1-T8) with three replications were performed, T_1_ was categorized as untreated control (healthy), while T_2_ treatment was inoculated with both *T. harzianum* MC2 and *T. harzianum* NBG. Additionally, T_3_ and T_4_ treatments were inoculated separately by *T. harzianum* MC2 and *T. harzianum* NBG. T_5_ was inoculated with only pathogen *R. solanacearum*, while T_6_ and T_7_ were treated with *R. solanacearum* along with *T. harzianum* MC2 and *T. harzianum* NBG, respectively. Treatment T_8_ was inoculated with *R. solanacearum* and both the bioagents *T. harzianum* MC2 and *T. harzianum* NBG. Both the pathogen and bioagents were applied after 10 days of transplanting in 3-week-old seedlings. Disease incidences and agronomic characteristics like root and shoot length, number of leaves, number of fruits per plant, and yield were evaluated. The formula used for disease incidence was:


$$Disease\;incidence\;(\%)=\frac{No\;of\;diseased\;plants}{total\;no\;of\;plants}\;x\;100$$

#### 2.4.1 Root colonization studies by electron microscopy

Seven-day**-**old tomato seedlings were used for scanning electron microscopy studies. The seedlings were initially treated with *Ralstonia solanacearum* for 15 mins followed by treatment with *T. harzianum* MC2 and *T. harzianum* NBG. Again, another set of seedlings was maintained as a control by being treated only with *Ralstonia solanacearum*. They were planted in sterilized soil and kept in sterile conditions. After seven days, seedlings from both treatment and control were randomly selected and subjected to electron microscopy studies. Tomato seedling roots were fixed in 2% glutaraldehyde (made up in a 0.1 M cacodylate buffer) in the refrigerator (8°C) for 1.5 hr. Samples were washed twice in the same buffer for 10 min, postfixed in 1% OsO4 for 4 hrs, and dehydrated as follows: 30%, 50%, 70%, 85%, and 95% ethanol for 15 min; 100% ethanol, twice for 15 min each. The Critical Point Drying (CPD) method, platinum coating, and an Amray 1600 scanning electron microscope operating at 20 kv were used for the scanning electron microscopy.

### 2.5 Statistical analysis

The experiment was designed in a randomized block design (RBD) comparing the treatments using critical difference (CD) at a 5% level of significance. A total of 8 treatments were maintained in 3 replications. The data were subjected to analysis of variance (ANOVA) using an online statistical analysis package (OPSTAT, Computer Section, CCS Haryana Agricultural University, Hisar 125004, Haryana, India).

## 3 Results

### 3.1 Isolation and screening of rhizospheric microbes

A total of five hundred and eighty (580) rhizospheric microbes were isolated from the one hundred and forty-five (145) rhizosphere soil samples of three solanaceous crops: tomato, brinjal, and chilli. Details regarding the agro-climatic zone, latitudes, and longitudes of soil samples are mentioned in **Supplementary Table S1**. Among the 580 rhizospheric microbes screened for antagonistic activity, 25 of the isolates demonstrated moderate to strong inhibition antagonistic effects against bacterial pathogen *R. solanacearum*, and three fungal pathogens, *R. solani*, *F. oxysporum f.sp. lycopersicum* and *C. gloeosporioides*, listed in **Supplementary Table S2**. The table depicts that out of the 25 active isolates, eight isolates, *viz*., JC1, JC3, MCG, MC2, NBG, NBY, NC1, and NC2, showed more than 80% activity against all three fungal pathogens, and greater than 35% antagonistic activity against bacterial pathogens. These isolates were further selected for morpho-molecular identification and detailed secondary screening comprehending antagonistic, biochemical, metabolomics, and *in planta* studies.

### 3.2 Identification of the potent rhizospheric microbes

**Figure 1:**
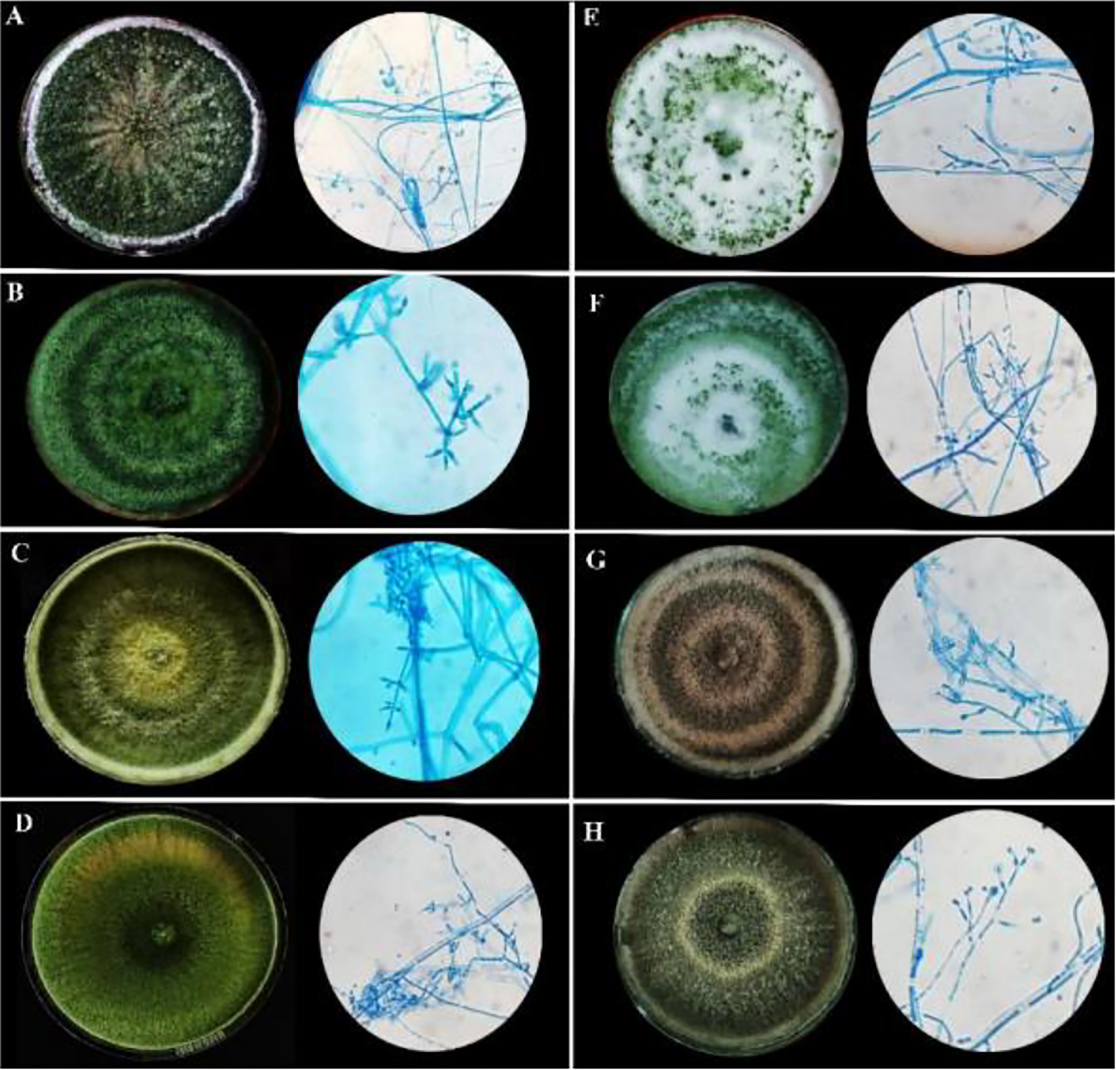
Morphological characteristics of rhizospheric fungal colonies on PDA media after sporulation with a detailed study of conidiophore, conidia, and phialides under 40X light microscope. **(A)** MC2 **(B)** NBG **(C)** NBY **(D)** MCG **(E)** JC1 **(F)** JC3 **(G)** NC1 **(H)** NC2.

Under a microscope, the vegetative hyphae were hyaline and septate with a smooth wall. Conidiophores were highly branched and primary branches were usually formed at nearly 90° to the main axis and lateral branches were more or less uniformly spaced. The phialides were flask-shaped, formed at the tips of the branches in verticillate whorls. Conidia were one-celled, globose in shape, and green-colored with a smooth surface. All eight microbes produced chlamydospores after 5-7 days. Chlamydospores were unicellular, globose in shape formed at the tip of the hypha or within the hyphae. Based on the morphological characteristics of the isolates, all eight promising rhizospheric microbes were tentatively identified as *Trichoderma* (Rifai, 1969). No major differentiating variations in the morphological characters had been observed among the eight rhizospheric isolates as depicted in **Table 1A** and **Figure 1**.

**Table 1:** Morpho-cultural characterization of the eight bioagents.

A. Morphological Identification			
Sl. No.	Morphological Feature	Characteristics	Strains
1	Conidial shape	Ellipsoidal subglobose	MC2, NBG, JC1, JC3, NC1, NC2, MCG, NBY
2	Phialides shape	Bowling pin, lageniform	MC2, NBG, JC1, JC3, NC1, NC2, MCG, NBY
3	Phialide disposition	Tending clusters, Spores produced successively at tip of phialides forming globose glocoid head	MC2
		Tending clusters	NBG, JC1, JC3, NC2
		Single	NC1, MCG, NBY
4	Chlamydospores	Formed at tips	MC2
		Terminal and intercalary	NBG, JC1
		Terminal	JC3, NBY
		Frequent and terminal	NC1, NC2, MCG
5	Conidiophore branching	Conifer and verticillate	MC2, NBG, JC1, JC3, NC1, NC2, MCG, NBY
6	Hyphae color and septation	Hyaline and septate	MC2, NBG, JC1, JC3, NC1, NC2, MCG, NBY
B. Culture Identification			
Sl. No.	Cultural variability Features	Characteristics	Strains
1	Growth rate (90mm)	5 days	MC2
		6 days	NBG
		7days	JC1, JC3, NC1, NC2, MCG, NBY
2	Texture	Dense and granular	MC2
		Dense hairy	NBG, NC1, NC2, NBY
		Cottony growth	JC1, JC3
		Fine granular	MCG
3	Sporulation	Excellent sporulation	MC2, NBG, NC1, NC2, NBY
		Moderate sporulation	MCG
		Low sporulation	JC1, JC3
4	Colony color	Deep olive green with radial sporulation	MC2
		Bright and dark green concentric rings	NBG
		White mat with very less patch of sporulation	JC1, JC3
		Distinct light and slightly fade olive green concentric rings	NC1
		Deep olive green with radial sporulation	MC2
		Distinct light and deep olive-green concentric rings	NC2
		Bright green with no concentric rings	MCG
		Bright green and yellowish concentric rings	NBY
5	Colony reverse color	White	MC2, NBG, JC1, JC3, NC1, NC2, MCG, NBY

The cultural characterization of the eight *Trichoderma* spp. was done on Potato Dextrose Agar media (PDA). The rate of mycelial growth of the isolates MC2 (**Figure 1A**) and NBG (**Figure 1B**) was highest compared to the other isolates. Dense mycelia and high sporulation were observed in the isolates MC2, NBG, and NC1. A moderate amount of sporulation was seen in MCG (**Figure 1D**), NC1 (**Figure 1G**), and NC2 (**Figure 1H**). A slower and low amount of sporulation was observed in the isolates JC1 (**Figure 1E**) and JC3 (**Figure 1F**). In terms of culture color, a dark green culture mat was observed in isolate MC2 while bright medium green mats were observed in isolates NC1, NC2, NBG, and MCG. Olive green culture mat with concentric rings was observed in NC1. White mycelial mat with green sporulation zones was observed in JC1 and JC3. Detailed cultural characterization is included in **Table 1B**.

**Figure 2:**
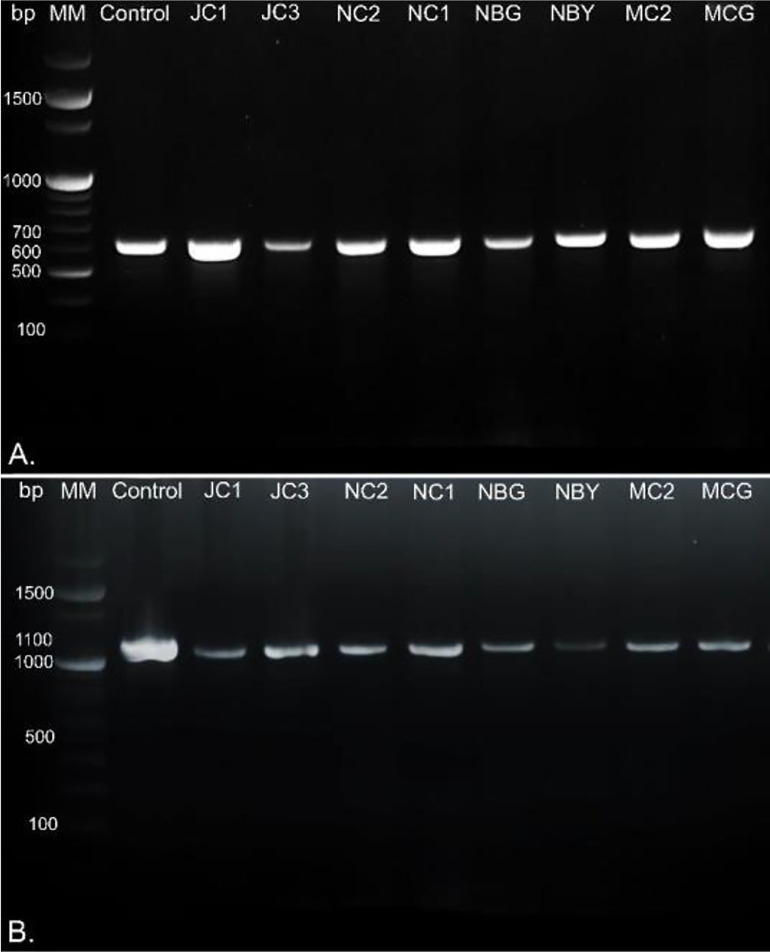
DNA gel electrophoresis profile generated by **(A)** ITS and **(B)** *tef1α* primers with rhizospheric fungal isolates with 100bp marker, MM, Molecular Marker; Control, Positive control; bp, base pairs.

**Figure 3:**
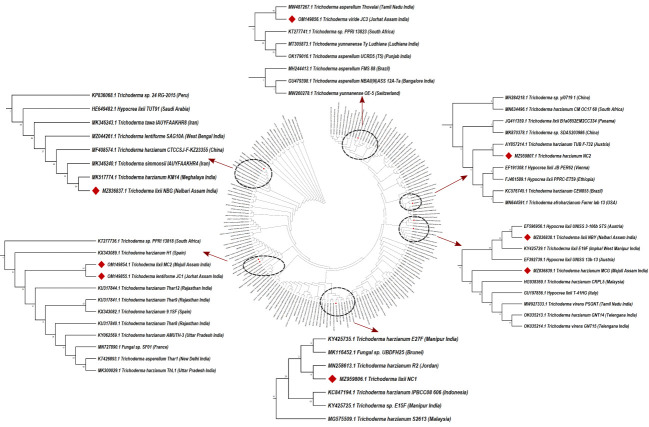
Phylogenetic tree of representative isolates of Trichoderma spp. inferred by Maximum Parsimony analysis of ITS1, 5.8s and ITS2 sequences in MEGA X11. The bootstrap support from 500 replications is indicated on the branches.

Morpho-cultural characterization was followed by molecular DNA sequencing. The ITS and *tef1α* regions were successfully amplified by PCR. PCR amplification of ITS region and *tef1α* produced the expected size of PCR product showing bands at around 650bp and 1150bp, respectively. Band patterns on agarose gel electrophoresis of ITS and *tef1α* PCR products of all the eight isolates are shown in **Figures 2A, B**. Sequence identification based on BLASTn (Nucleotide BLAST) and phylogenetic analysis are summarized in **Figure 3**, **4** which confirms the strains as *Trichoderma* spp. The accession IDs of the eight strains were retrieved from NCBI GenBank. All eight sequences are identified as *Trichoderma* spp. (**Table 2**). Specimens of *Hypocrea. lentiformis, H. lixii*, and *H. harzianum* show no morphologically distinguishing characters from each other and therefore represent one species. *H. lixii* is the oldest name and hence is considered the correct name for these species (Chaverri and Samuels, 2002; Dodd et al., 2002).

**Figure 4:**
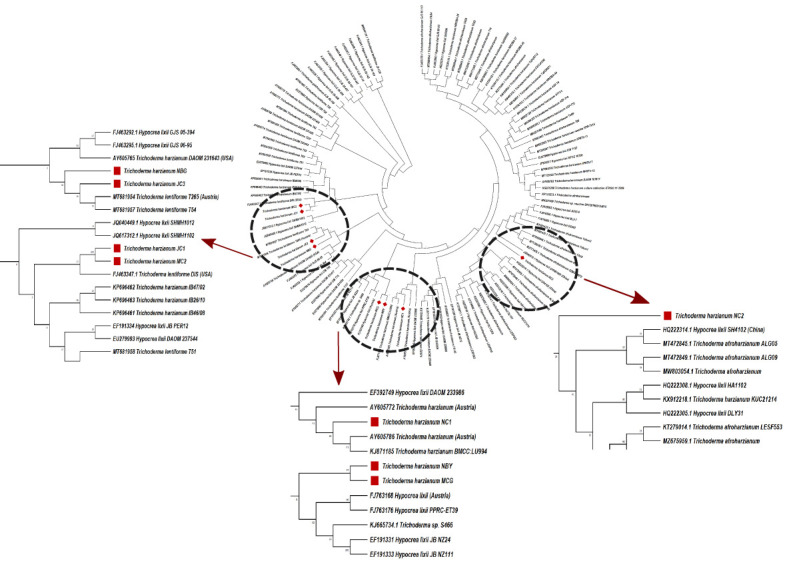
Phylogenetic tree of representative isolates of Trichoderma spp. inferred by Maximum Parsimony analysis of Tef-1-α sequences in MEGA 4.0. The bootstrap support from 500 replications is indicated on the branches.

**Table 2:** Molecular identification of ITS and *tef 1 α* based molecular markers.

A. ITS based Identification		
Isolate ID	Species Identified	NCBI Accession ID
MC2	*Trichoderma lixii* MC2	OM149854
JC1	*Trichoderma lentiforme* JC1	OM149855
JC3	*Trichoderma viride* JC3	OM149856
NC1	*Trichoderma lixii* TH_ITS1	MZ959806
NC2	*Trichoderma harzianum* TV_ITS1	MZ959807
NBG	*Trichoderma lixii* NBG	MZ836837
NBY	*Trichoderma lixii* NBY	MZ836838
MCG	*Trichoderma harzianum* MCG	MZ836839
B. *Tef* 1 *α* based Identification		
Isolate ID	Species Identified	NCBI Accession ID
NC2	*Trichoderma harzianum* NC2	OP313506
NBG	*Trichoderma harzianum* NBG	OP313507
NC1	*Trichoderma harzianum* NC1	OP313508
MC2	*Trichoderma harzianum* MC2	OP313509
JC1	*Trichoderma harzianum* JC1	OP313510
JC3	*Trichoderma harzianum* JC3	OP313511
NBY	*Trichoderma harzianum* NBY	OP313512
MCG	*Trichoderma harzianum* MCG	OP313513

### 3.3 Antimicrobial activity of the potent rhizospheric microbes

#### 3.3.1 Inhibition of radial growth of phytopathogens

**Figure 5:**
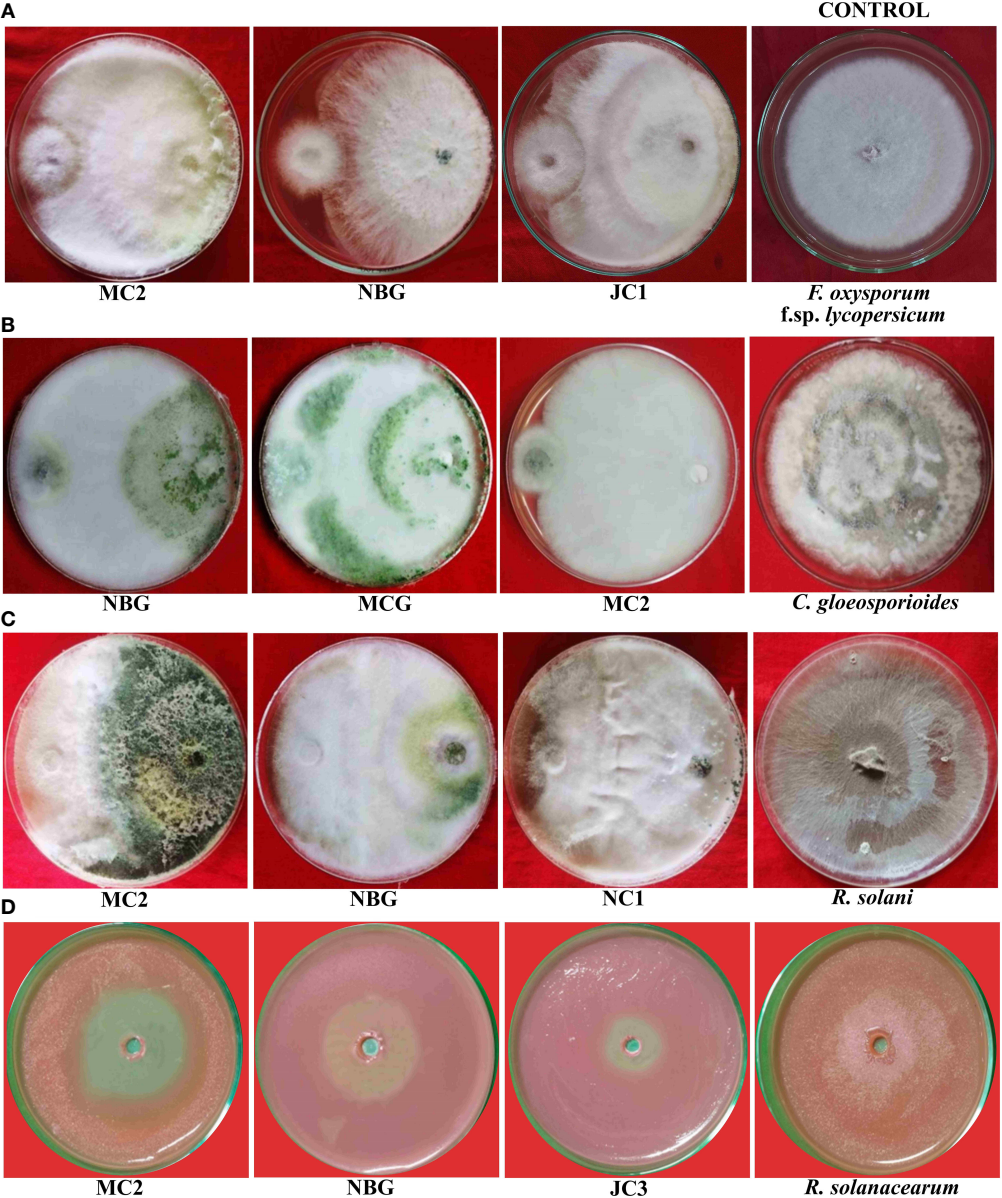
Antagonistic effects of rhizospheric isolates against four plant pathogens, **(A)** *Fusarium oxysporum* f sp. *lycopersicum*, **(B)** *Colletotrichum gleoesporioides*, **(C)** *Rhizoctonia solani*, **(D)** *Ralstonia solanacearum* on solid PDA media after 6 days.

All eight fungal isolates in general impede ≥80% microbial growth of all the targeted fungal pathogens. **Table 3** and **Figure 5** show a comparative account of the percent inhibition of eight selected antagonistic microbes against the full growth control plate of the four test pathogens, in absence of any bioagent. Notably, isolates MC2 and NBG apparently surpass the other six isolates in terms of their antagonistic potential against the fungal and bacterial test pathogens.

**Table 3:** Antagonistic activity of the eight fungal isolates against pathogens causing diseases in solanaceous crops.

Treatment No.	Bioagents	*Fusarium oxysporum* f. sp. *lycopersicum*		*Colletotrichum gloeosporioides*		*Rhizoctonia solani*		*Ralstonia solanacearum*	
		Radial growth (mm)	Inhibition (%)	Radial growth (mm)	Inhibition (%)	Radial growth (mm)	Inhibition (%)	Radial growth (mm)	Inhibition (%)
T_0_	Control	90.00	00.00	90.00	0.00	90.00	0.00	90.00	0.00
T_1_	JC1	1.00	88.80	1.02	88.66	0.27	97.00	31.66	35.17
T_2_	JC3	1.50	83.60	0.95	89.44	0.50	94.44	35.66	39.62
T_3_	MCG	1.70	81.80	0.60	93.30	0.60	93.33	27.00	30.00
**T_4_**	MC2	0.07	92.50	0.77	91.44	0.00	100.00	46.00	51.00
**T_5_**	NBG	1.00	88.80	0.50	94.44	0.00	100.00	41.66	46.29
T_6_	NC2	1.70	80.00	1.02	88.66	1.00	88.88	34.80	38.66
T_7_	NC1	1.50	83.30	1.20	86.66	0.12	98.66	34.66	38.51
T_8_	NBY	1.70	80.00	1.02	88.66	0.80	91.11	31.33	34.81
**CD**		5.21		2.63		0.11		1.33	
**± SE(d)**		2.52		1.27		0.05		0.64	

CD, Critical Difference at 5% (p< 0.05), SE(d), Standard Error of Deviation.

Data recorded for inhibition against *F. oxysporum* f. sp. *lycopersicum* reveals maximum inhibition at 92.50% exhibited by the isolate MC2, followed by the isolates NBG and JC1, showing 88.80% inhibitory activity equivalently (**Table 3**; **Figure 5A**). Notably other isolates MCG, JC3, NC1, NC2, and NBY also inhibit *Fusarium* activity within a range of 80% – 83%, as shown in **Supplementary Figure S1** in Appendix. Three isolates, NBG, MCG, and MC2 depicted the highest inhibition against *C. gloeosporioides*, shown in **Figure 5B**. **Table 3** shows that the highest mycelial inhibition rate of 94.44% was recorded in the isolate NBG when confronted with the pathogen, followed by 93.30% and 91.44% shown by MCG and MC2, respectively. The remaining isolates displayed inhibition rates above 85%. The antagonistic ability of eight isolates against *C. gloeosporioides* is summarized in **Supplementary Figure S2**. The antagonism potential of the eight strains was also evaluated against *R. solani* in **Figure 5C** and **Table 3**, which represents maximum and complete inhibition by isolates NBG and the isolate MC2 with 100% inhibition rate. Antagonism intensity measured as the inhibition percentage for NC1 and JC1 is calculated as 98.66% and 97.00%, respectively, while the rest of the strains show better rates between 88% – 94%. **Supplementary Figure S3** gives a representation of all eight isolates against *R. solani.*

The maximum suppression measured in terms of the highest zone of inhibition of the pathogen *R. solanacearum* was recorded in the isolate MC2 (51.00%) followed by the isolate NBG (46.29%). The rest of the isolates also exhibited zone of inhibition over 30 percent against the growth of the pathogen (**Figure 5D**, **Table 3**).

#### 3.3.2 Effect of volatile compounds on target pathogens

**Figure 6:**
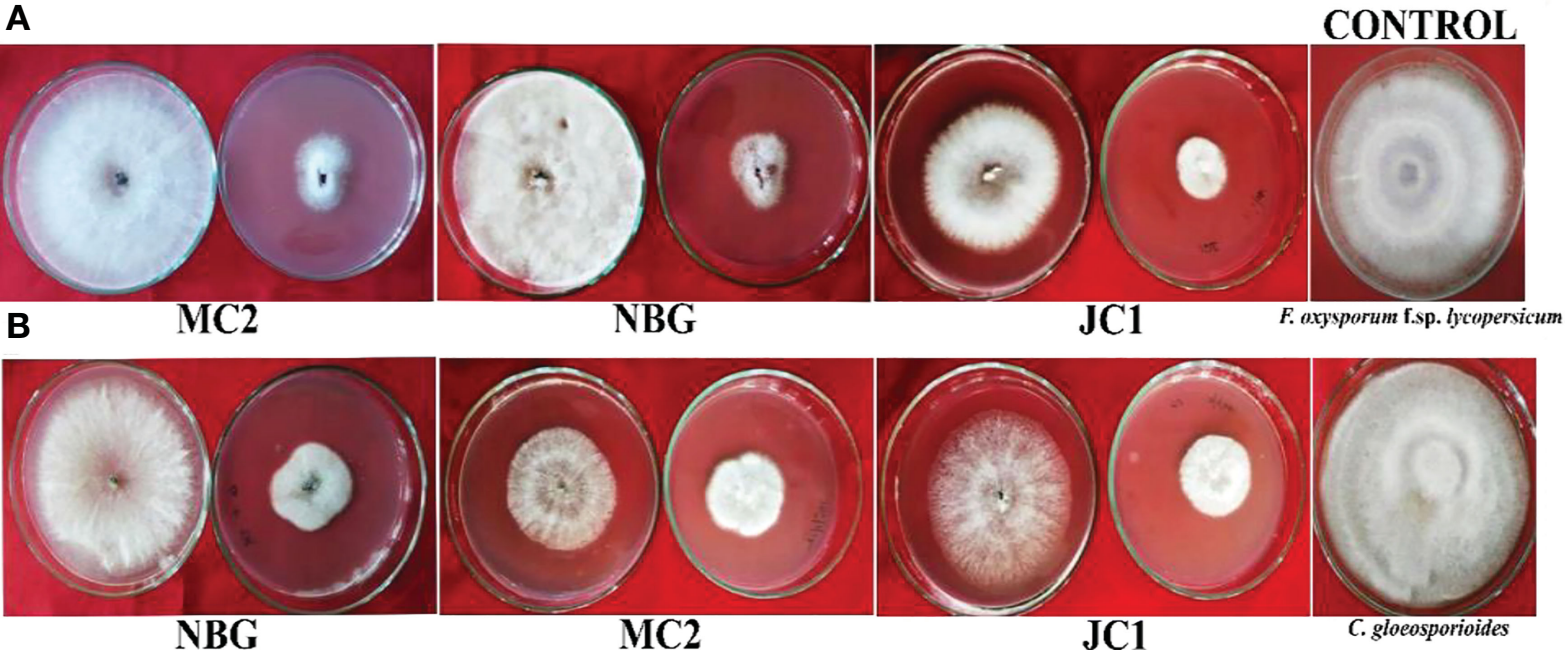
Dual culture assay for bioactivity of volatile organic compounds of the rhizospheric isolates on plant pathogens: **(A)** *Fusarium oxysporum* f.sp. *lycopersicum*, **(B)** *Colletotrichum gleoesporioides.* The bioagent culture was on the bottom and the test pathogen culture was on the lid facing downwards. Both the bioagent and pathogen were grown on PDA and exposed to each other for 7 days.

To further justify the antagonistic ability of the eight microbes, the antifungal and antibacterial effects of volatile compounds produced by the eight promising rhizospheric microbes were investigated *in vitro*. The results of the inhibition of the pathogen growth by the isolates are summarized in **Table 4** and **Figure 6**. The highest percent inhibition was recorded in the isolate MC2 (77.11%) followed by NBG (75.55%) and JC1 (66.66) against the radial mycelial growth of *F. oxysporum* f. sp. *lycopersicum*, which were significantly superior to percent inhibition exhibited by the other isolates, clearly shown in **Table 4** and graphically represented by **Figure 6A**. **Figure 6B** shows a limited growth of *C. gloeosporioides* attributed to the presence of volatile compounds in the isolates. Maximum inhibition of 73.33% was observed in the isolate NBG, followed by 72.22% in the isolate MC2, shown in **Table 4**. Pictorial representations of all the eight isolates inhibiting the two test pathogens are compiled in **Supplementary Figures S5** and **S6** for *F. oxysporum* f. sp. *lycopersicum* and *C. gloeosporioides* respectively. No significant inhibition was observed in any of the isolates against *R. solani*.

**Table 4:** Effect of volatile compound production of promising rhizospheric microbes on fungal pathogens.

Treatment No.	Bioagents	*Fusarium oxysporum* f. sp. *lycopersicum*		*Colletotrichum gloeosporioides*	
		Radial growth (mm)	Inhibition (%)	Radial growth (mm)	Inhibition (%)
T_0_	Control	90.00	0.00	90.00	0.00
T_1_	JC1	3.00	66.66	3.66	59.33
T_2_	JC3	3.53	60.73	4.40	51.11
T_3_	MCG	3.33	63.00	4.03	55.22
**T_4_**	**MC2**	2.06	77.11	2.50	72.22
**T_5_**	**NBG**	2.20	75.55	2.40	73.33
T_6_	NC2	3.66	59.33	4.10	54.44
T_7_	NC1	3.03	66.33	4.33	51.88
T_8_	NBY	3.56	60.44	4.60	48.88
**CD**		0.29		0.23	
**± SE(d)**		0.13		0.11	

CD, Critical Difference at 5% (p< 0.05); SE (d), Standard Error of Deviation.

#### 3.3.3 Effect of non-volatile compounds on target pathogens

**Figure 7:**
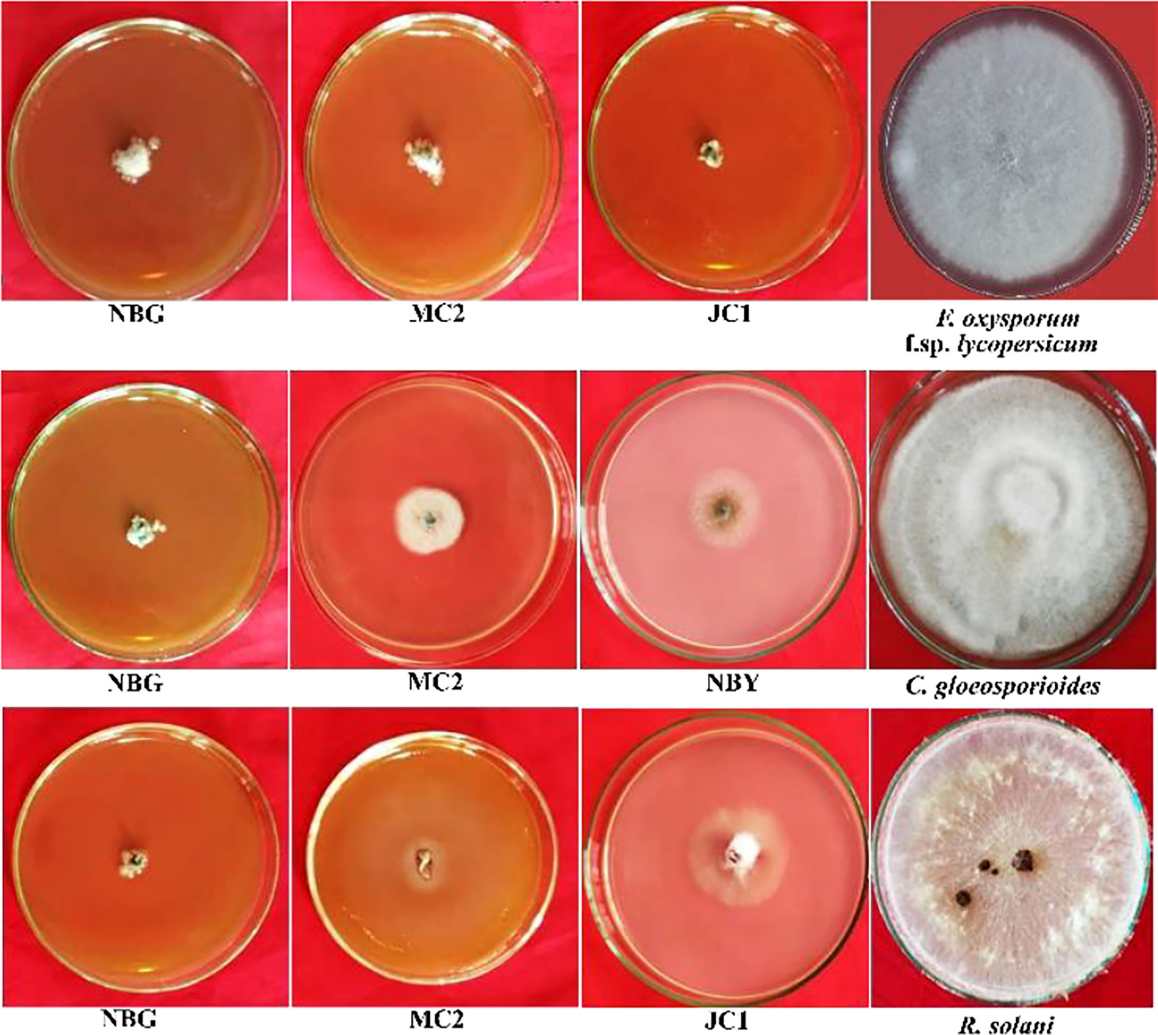
Effects of cultural filtrates of rhizospheric isolates on the growth of four plant pathogens: Fusarium oxysporum f sp. lycopersicum, Colletotrichum gleoesporioides, Rhizoctonia solani. The highest inhibition of the pathogens was observed at 50% concentration of culture filtrate with PDA media for fungal pathogens.

A study was conducted to investigate the non-volatile compounds production potential by the eight promising rhizospheric microbes; the three highest inhibitions against each of the pathogens are shown in **Figure 7**. The data reveals that the strains secreted non-volatile metabolites in all three concentrations into the liquid medium and the filtrates inhibited the growth of pathogens. Evaluation of produced non-volatile components showed noticeable performance on inhibiting the mycelial growth of pathogens at 50% concentration of the filtrates. The percent inhibition of all the pathogens was calculated and compared to the pathogen on obtaining its full growth in the control plate (**Table 5**). Against *R. solanacearum*, the highest inhibition zone was produced by the isolate MC2 (51.00%). Isolate NBG showed the highest inhibition against all three fungal pathogens with 95.55%, 97.00%, and 96.33% inhibition recorded against *F. oxysporum* f. sp. *lycopersicum, C. gloeosporioides*, and *R. solani*, respectively. On average, all the isolates showed acceptable inhibition, compiled in **Supplementary Figures S4**, **S7**–**S9**.

**Table 5:** Effect of non-volatile compound production of promising rhizospheric microbes on pathogens.

Treatment No.	Bioagents	*Fusarium oxysporum* f. sp. *lycopersicum*		*Colletotrichum gloeosporioides*		*Rhizoctonia solani*		*Ralstonia solanacearum*	
		Radial growth (mm)	Inhibition (%)	Radial growth (mm)	Inhibition (%)	Radial growth (mm)	Inhibition (%)	Radial growth (mm)	Inhibition (%)
T_0_	Control	90.00	0.00	90.00	0.00	90.00	0.00	90.00	0.00
T_1_	JC1	0.43	95.22	1.83	79.66	2.76	69.33	31.66	35.17
T_2_	JC3	1.63	81.88	1.86	79.33	3.46	61.55	35.66	39.62
T_3_	MCG	1.66	81.56	2.13	76.33	3.10	65.55	27.00	30.00
T_4_	MC2	0.30	96.00	1.40	84.44	2.56	71.55	46.00	51.00
T_5_	NBG	0.23	97.00	0.33	96.33	0.40	95.55	41.66	46.29
T_6_	NC2	2.23	75.27	3.16	64.88	3.00	66.66	34.80	38.66
T_7_	NC1	2.16	76.00	2.00	77.77	3.16	64.88	34.66	38.51
T_8_	NBY	2.03	77.44	1.70	81.11	4.16	53.77	31.33	34.81
CD		0.22		0.19		0.30		1.33	
± SE(d)		0.10		0.09		0.14		0.64	

CD, Critical Difference at 5% (p< 0.05); SE (d), Standard Error of Deviation.

#### 3.3.4 Qualitative assay of extracellular enzymes

**Figure 8:**
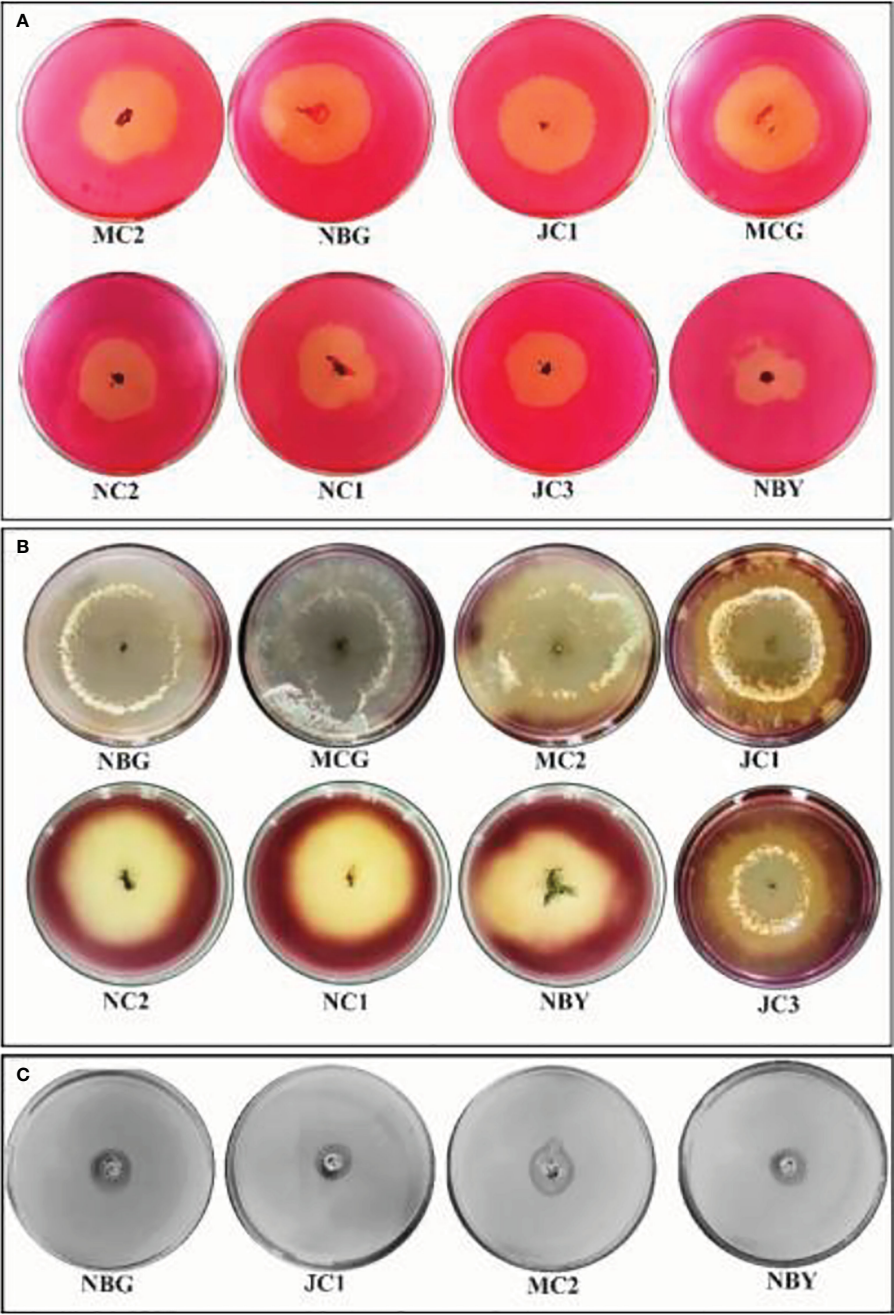
Zonations of fungal microbial colonies in qualitative plate assay showing the release of cellulase, amylase, and protease enzyme by rhizospheric isolates. **(A)** Cellulase **(B)** Amylase **(C)** Protease.

The eight promising rhizospheric microbes were tested for their ability to secrete extracellular enzymes against pathogens causing diseases in solanaceous crops. All eight fungal strains expressed relevant cellulolytic (**Figure 8A**) and amylase activity (**Figure 8B**) which was evident from the distinct yellow halo zone surrounding their colony growth portion (**Table 6**). The greater the zone the more the enzymes are secreted. However, proteolytic activity was expressed by four out of the eight promising rhizospheric microbes with a distinct opaque zone surrounding their colony (**Figure 8C**).

**Table 6:** Activity of the eight promising bioagents to secrete extracellular enzymes.

Bioagents	Cellulase	Amylase	Protease
**MC2**	**+**	**+**	**+**
**NBG**	**+**	**+**	**+**
**JC1**	**+**	**+**	**+**
**MCG**	**+**	**+**	–
**NC2**	**+**	**+**	–
**JC3**	**+**	**+**	**+**
**NC1**	**+**	**+**	–
**NBY**	**+**	**+**	–

+ positive reaction, – negative reaction.

#### 3.3.5 Analysis of antimicrobial compounds through LCMS

Antagonism of the isolates necessitated the exploration of possible antimicrobial compounds released by the isolates. Such a metabolomics study is relevant to clearly understand the antimicrobial behavior of the isolates. Based on the secondary screening to evaluate potential antimicrobial activities, two of the isolates, *T. harzianum* MC2 and *T. harzianum* NBG were further considered for identification of bioactive compounds *via* LCMS that may contribute towards developing the isolates as effective bioagents.

**Figure 9:**
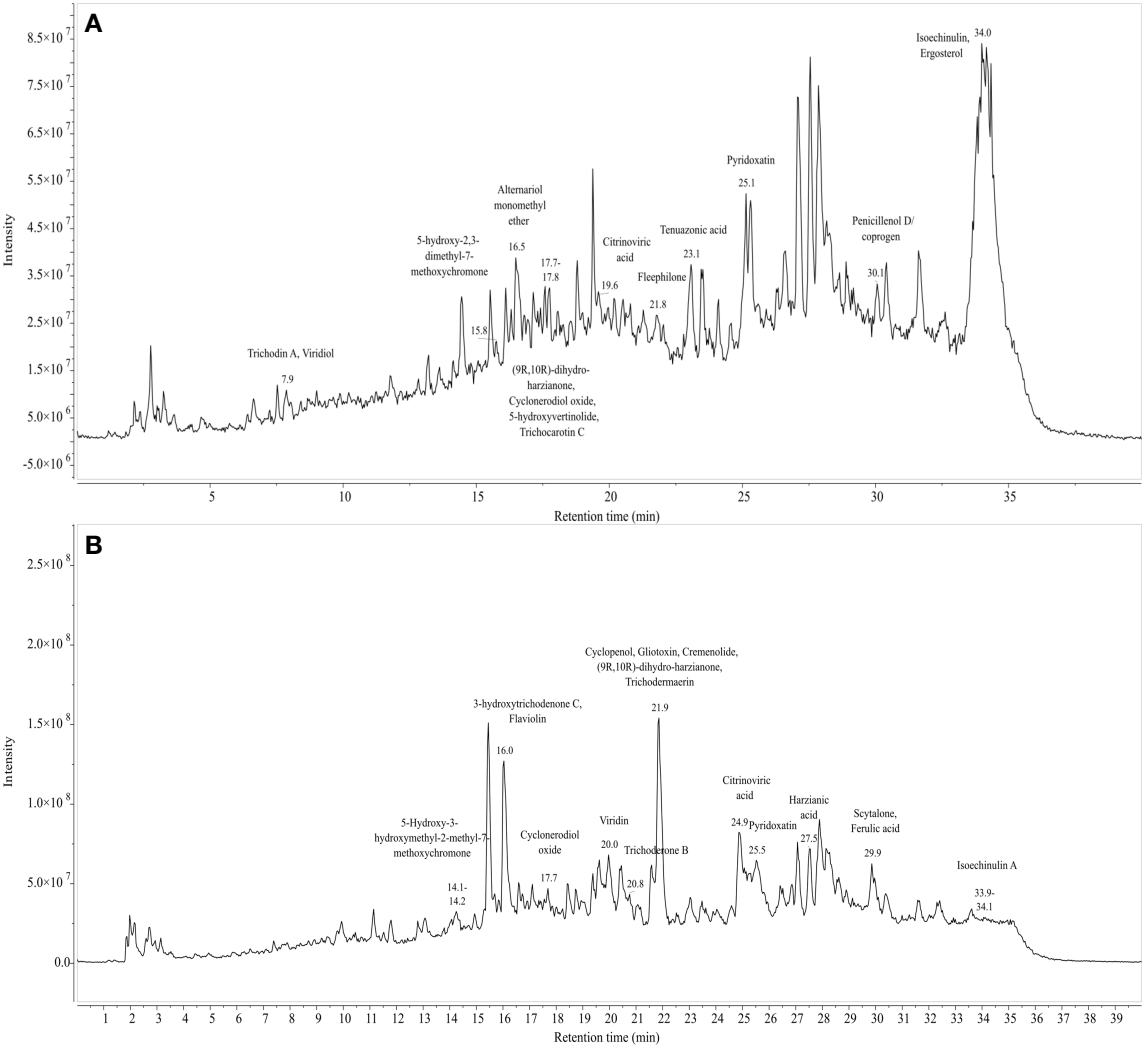
Total ion chromatogram of LC-MS-ESI of cell free filtrates of *Trichoderma* isolates which outstood the *in vitro* antagonistic assays. **(A)** NBG **(B)** MC2.

**Figures 9A**, **9B** represent the total ion chromatogram (TIC) for metabolites from cell-free supernatants of *T. harzianum* MC2 and *T. harzianum* NBG, respectively. It is clearly indicative of the presence of various active metabolites in methanol: chloroform crude extract of *T. harzianum* MC2 and *T. harzianum* NBG, respectively. **Tables 7A, B** enlisted 15 and 17 compounds from the extract of *T. harzianum* MC2 and *T. harzianum* NBG, respectively, indicated by their chromatographic and spectral characteristics identified by Mestrenova (MNOVA). Individual mass spectra of all the compounds are compiled in **Supplementary Figures S10**, **S11**.

**Table 7:** List of antimicrobial compounds retrieved from LCMS analysis of *T. harzianum* MC2 and *T. harzianum* NBG.

A. Significant metabolites derived from LCMS extract of *T. harzianum* NBG								
Sl.no	Molecule	Formula	Match score	Similarity	Retention time	Molecule Class	Function	Reference
1	Trichodin A	C_21_H_25_NO_3_	0.982	0.982	7.87	Pyridine	Antibacterial, Antifungal	(Wu et al., 2014)
2	Viridiol	C_20_H_18_O_6_	0.986	0.986	7.87	Steroid	Antifungal	(Moffatt et al., 1969)
3	5-hydroxy-2,3-dimethyl-7-methoxychromone	C_12_H_12_O_4_	0.88	0.88	15.76	Ketone	No obvious function	
4	Alternariol monomethyl ether	C_15_H_12_O_5_	0.879	0.879	16.52	Ether	Mycotoxin	(Demuner et al., 2013)
5	(9R,10R)-dihydro-harzianone	C_20_H_32_O	0.914	0.914	17.73	Diterpene	Cytotoxic	(Ortega et al., 2021)
6	Cyclonerodiol oxide	C_15_H_28_O_3_	0.907	0.907	17.73	Sesquiterpene	Plant growth regulation	(Cutler et al., 1991)
7	5-hydroxyvertinolide	C_14_H_18_O_5_	0.967	0.967	17.76	Butenolide	Antifungal	(Vinale et al., 2009b)
8	Trichocarotin C	C_15_H_22_O_3_	0.955	0.955	17.76	Sesquiterpene	Antimicroalgal	(Shi et al., 2018)
9	Citrinoviric acid	C_14_H_20_O_6_	0.966	0.966	19.69	Polyketides	Cytotoxic	(Zhang et al., 2014)
10	Fleephilone	C_24_H_27_NO_7_	0.876	0.876	21.72	Azaphilones	No obvious activity	(Qian-Cutrone et al., 1996)
11	Tenuazonic acid	C_10_H_15_NO_3_	0.95	0.95	23.1	Tetrameric acid	Phytotoxin	(Yun et al., 2015)
12	Pyridoxatin	C_15_H_21_NO_3_	0.949	0.949	25.14	Dihydroxypyridines	Antifungal, Antibiotic	(Wu et al., 2014)
13	Penicillenol D/Coprogen	C_17_H_29_NO_4_	0.88	0.88	30.06	Hydroxamate siderophores	Cytotoxic	(Anke et al., 1991)
14	Isoechinulin A	C_24_H_29_N_3_O_2_	0.948	0.948	33.99	Alkaloids	Antialgal	(Shi, 2018)
15	Ergosterol	C_28_H_44_O	0.875	0.875	33.99	Sterol	Antifungal	(Tarus et al., 2003)
B. Significant metabolites derived from LCMS extract of *T. harzianum* MC2								
Sl.no	Molecule	Formula	Match score	Similarity	Retention time	Molecule class	Function	References
1	5-Hydroxy-3-hydroxymethyl-2-methyl-7-methoxychromone	C_12_H_12_O_5_	0.995	0.995	14.07	Ketone	No obvious activity	
2	3-hydroxytrichodenone C	C_7_H_9_ClO_3_	0.946	0.946	16.1	Ketone	Antibacterial, Growth Inhibiting	(Song et al., 2018)
3	Flaviolin	C_10_H_6_O_5_	0.916	0.916	16.1	Naphthoquinone	No obvious activity	(Astill and Roberts, 1953)
4	Cyclonerodiol oxide	C_15_H_28_O_3_	0.88	0.88	17.69	Sesquiterpene	Plant growth regulation	(Cutler et al., 1991)
5	Viridin	C_20_H_16_O_6_	0.873	0.873	19.93	Steroidal antibiotics	Antifungal	(Brian and McGowan, 1945)
6	Trichoderone B	C_24_H_35_NO_5_	0.938	0.938	20.65	Cytochalasans	Cytotoxic	(Ding et al., 2012)
7	Cyclopenol	C_17_H_14_N_2_O_4_	0.914	0.914	21.86	Alkaloid	Antibacterial	(Shi, 2018)
8	Gliotoxin	C_13_H_14_N_2_O_4_S_2_	0.908	0.908	21.86	Diketopiperazines	Antifungal	(Singh et al., 2005)
9	Cremenolide	C_16_H_22_O_7_	0.998	0.998	21.86	Lactone	Antifungal and PGP	(Vinale et al., 2016)
10	(9R,10R)-dihydro-harzianone	C_20_H_32_O	0.899	0.899	21.9	Diterpenes	Cytotoxic	(Ortega et al., 2021)
11	Trichodermaerin	C_20_H_28_O_3_	0.943	0.943	21.9	Diterpenes	No obvious activity	(Chantrapromma et al., 2014)
12	Citrinoviric acid	C_14_H_20_O_6_	0.977	0.977	25.03	Polyketides	Cytotoxic	(Zhang et al., 2014)
13	Pyridoxatin	C_15_H_21_NO_3_	0.968	0.968	25.07	Dihydroxypyridines	Antibiotic	(Wu et al., 2014)
14	Harzianic acid	C_19_H_27_NO_6_	0.917	0.917	27.51	Tetramic acid derivatives	PGP	(Vinale et al., 2009a)
15	Scytalone	C_10_H_10_O_4_	0.99	0.99	29.86	Lyases	No obvious activity	(Zhang et al., 2017)
16	Ferulic acid	C_10_H_10_O_4_	0.99	0.99	29.86	Hydroxycinnamic acids/phenol	No obvious activity	(Yu et al., 2002)
17	Isoechinulin A	C_24_H_29_N_3_O_2_	0.928	0.928	33.86	Alkaloid	Antimicroalgal	(Shi, 2018)

*Ferulic acid, Scytalone, Harzianic acid, Cremenolide, viridian, 3-hydroxytrichodenone C.

**Figure 10:**
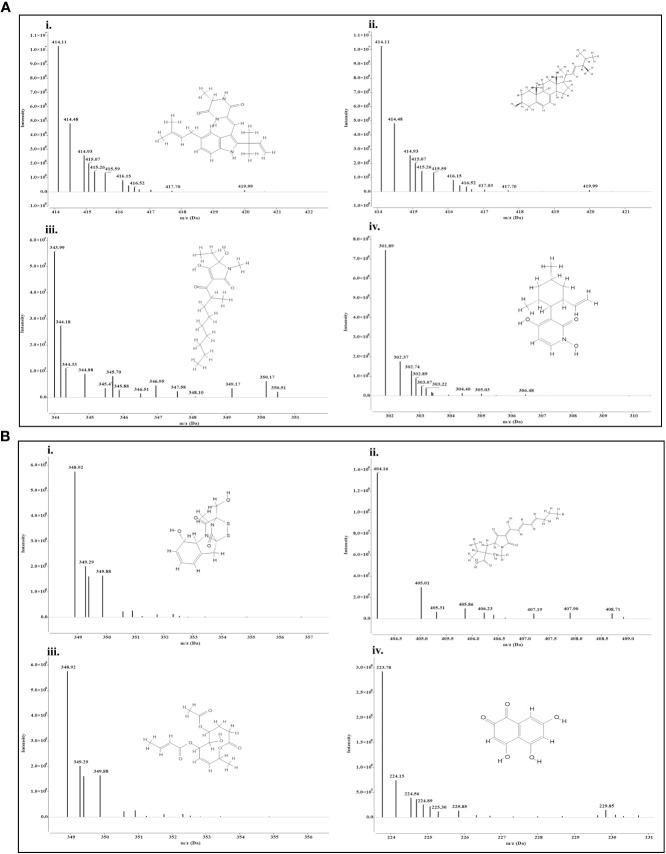
Mass spectra and chemical structure of four abundantly found antimicrobial compounds in **(A)** *Trichoderma harzianum* NBG - i) Isoechinulin A, ii) Ergosterol, iii) Coprogen D, and iv) Pyridoxatin. **(B)** *Trichoderma harzianum* NBG - i) Gliotoxin, ii) Harzianic acid, iii) Cremenolide D, and iv) Flaviolin.

Both the isolates released a set of compounds that can be broadly distinguished as diterpenes, sesquiterpene, butenolide, polyketide, alkaloid, ketones, and several other classes of molecules. A marked diversity of the secondary metabolites can be observed in both the bioagents which may correspond to their widely shown antagonisms. **Tables 7A, B** represent the compounds along with their chemical formulae, retention time, match score, similarity indices, and the known functions of the compounds. Non-volatile antifungal substances such as gliotoxin, cremenolide, viridin are reported from the LCMS extract of NBG. Trichodin A, viridiol, and 5-hydroxyvertinolide are some compounds that are retrieved from the extract of MC2. **Figures 10A**, **10B** illustrate the LCMS spectra of molecules with the highest abundance in the extracts of the two *Trichoderma* isolates.

#### 3.3.6 Biocontrol assessment of *T. harzianum* MC2 and *T. harzianum* NBG in controlling bacterial wilt in field experiments

**Figure 11:**
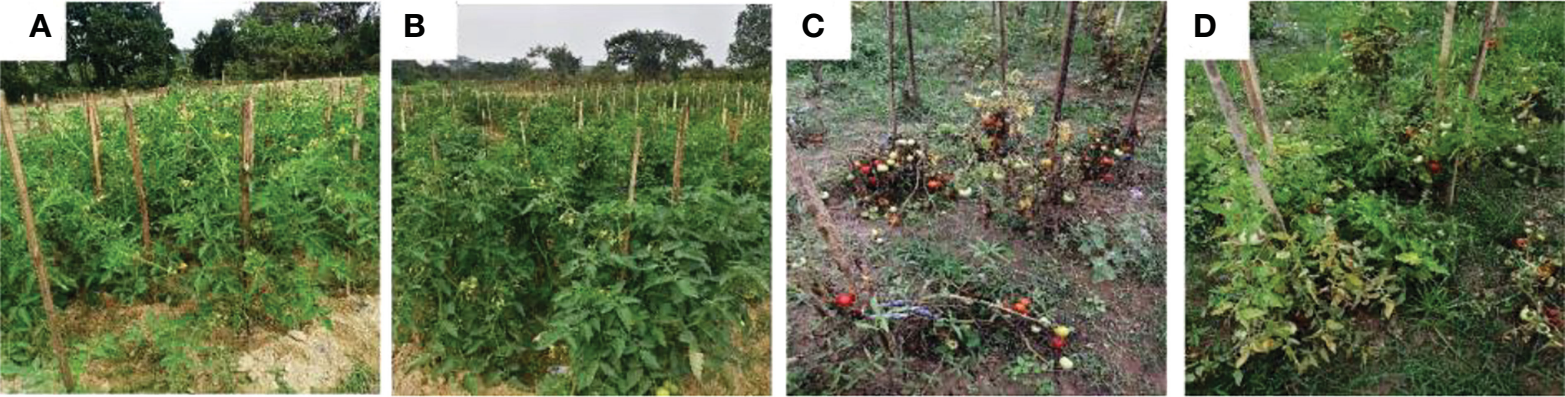
Biocontrol of bacterial wilt of tomato under field conditions using *Trichoderma harzianum* MC2 and *Trichoderma harzianum* NBG. **(A)** (T1) = Untreated control **(C)** (T5) = plants inoculated with *Ralstonia solanacearum* **(B)** (T2) = plants treated with both bioagents *Trichoderma harzianum* MC2 + *Trichoderma harzianum* NBG **(D)** (T8) = plants treated with *Trichoderma harzianum* MC2 + *Trichoderma harzianum* NBG and challenged with *Ralstonia solanacearum*.

Post *in vitro* biochemical and metabolomics analysis, the two most potential isolates *T. harzianum* MC2 and *T. harzianum* NBG retrieved from the rhizosphere soil of brinjal and chilli were assessed as biocontrol agents to control *R. solanacearum* in tomato plants. Tomato variety Pusa Ruby was used in the field experiment. The field evaluations were performed in Orchard, Assam Agricultural University, Jorhat. The first field experiment was done in the first week of December 2021 and the second field experiment was done in the third week of November 2022. The factorial design followed was Randomized Block Design with 8 treatments and 3 replications. Thirty beds of dimensions 2.4m x 2.0m were prepared maintaining the spacing of 0.6m row to row. In each bed, 16 seedlings were transplanted. In both years, the final harvest was done in the fourth week of the fourth month and subsequently, final data was taken along with it. The results are summarized in **Table 8** and **Figures 11A–D**. The results achieved were similar to laboratory assays.

**Table 8:** Disease incidence and growth parameters of eight treatments at harvest.

Treatment No.	Treatment Description	Disease incidence (%)	Shoot length (cm)	No of branches/plant	No of fruits/plant	Yield (Kg/plant)	Shoot dry weight (gram/plant)	Root dry weight (gram/plant)
T_1_	Untreated control	0.00	66.33	31.33	32.00	3.26	35.00	15.33
T_2_	MC2^*^ + NBG^†^	0.00	74.66	34.66	40.33	4.45	37.66	16.66
T_3_	*MC2*	0.00	72.33	34.66	35.66	3.78	31.66	13.00
T_4_	*NBG*	0.00	68.66	24.66	32.66	3.53	26.33	12.00
T_5_	Inoculated control (*RS^+^*)	100	41.00	16.66	18.66	1.67	15.33	02.66
T_6_	*NBG+RS*	77.50	68.55	24.66	32.66	3.53	26.33	12.00
T_7_	*MC2+RS*	61.50	72.33	34.66	35.66	3.78	31.66	13.00
T_8_	*MC2 + NBG* + *RS*	52.50	65.66	25.00	33.33	3.67	26.33	11.00
CD at 5%			1.91	2.34	4.95		2.41	3.13
± SE (m)			0.62	0.76	1.61		0.79	1.02

^*^ Trichoderma harzianum MC2, ^†^Trichoderma harzianum NBG, ^+^Ralstonia solanacearum, CD, Critical Difference at 5% (p< 0.05), SE (m), Standard Error of Mean.

In a set of eight treatments shown in **Table 8**, no disease incidences were recorded in untreated plots (under treatment T_1,_ **Figure 11A**), under the individual applications of *T. harzianum* NBG (treatment T_3_) and *T. harzianum* MC2 (treatment T_4_) as well as combined treatment T_2_ which includes both *T. harzianum* MC2 and *T. harzianum* NBG (**Figure 11B**). Furthermore, the biocontrol efficacy of the two bioagents was assessed when inoculated alongside *R. solanacearum* (**Table 8**). There was a 100% incidence of wilt upon introduction of *R. solanacearum* (treatment T_5,_ **Figure 11C**) which was lowered to 61-77% when inoculated with NBG (T_6_) and MC2 (T_7_) isolates. A significantly reduced wilt incidence of 52.50% was recorded in the plot treated with both *T. harzianum* MC2 and *T. harzianum* NBG (T_8_, **Figure 11D**) compared to other treatments. These results are clearly in agreement with the biocontrol potential of the two isolates for efficiently controlling bacterial wilt. In the combination treatment T_8_ (*T. harzianum* MC2 and *T. harzianum* NBG), a noticeable rise in the growth parameters and yield was observed as compared to inoculated control plot T_5_ (bacterial wilt infected). An increment of 54.49% in yield and a reduction of 47.50% in disease incidence was observed in the treatment T_8_ in comparison with the control. Shoot length, number of branches and fruits per plant, yield, and dry weight of root and shoot were also measured. On average, application of treatment T_8_ showed maximum overall increase in shoot length (mean = 74.66cm), number of branches (34.66cm), fruits (mean = 40.33/plant), dry shoot and root weights measuring 37.66g and 16.66g, respectively, at the time of final harvest. These results suggest that both isolates have the potential to increase tomato plant growth.

#### 3.3.7 Scanning electron microscopic observations

**Figure 12:**
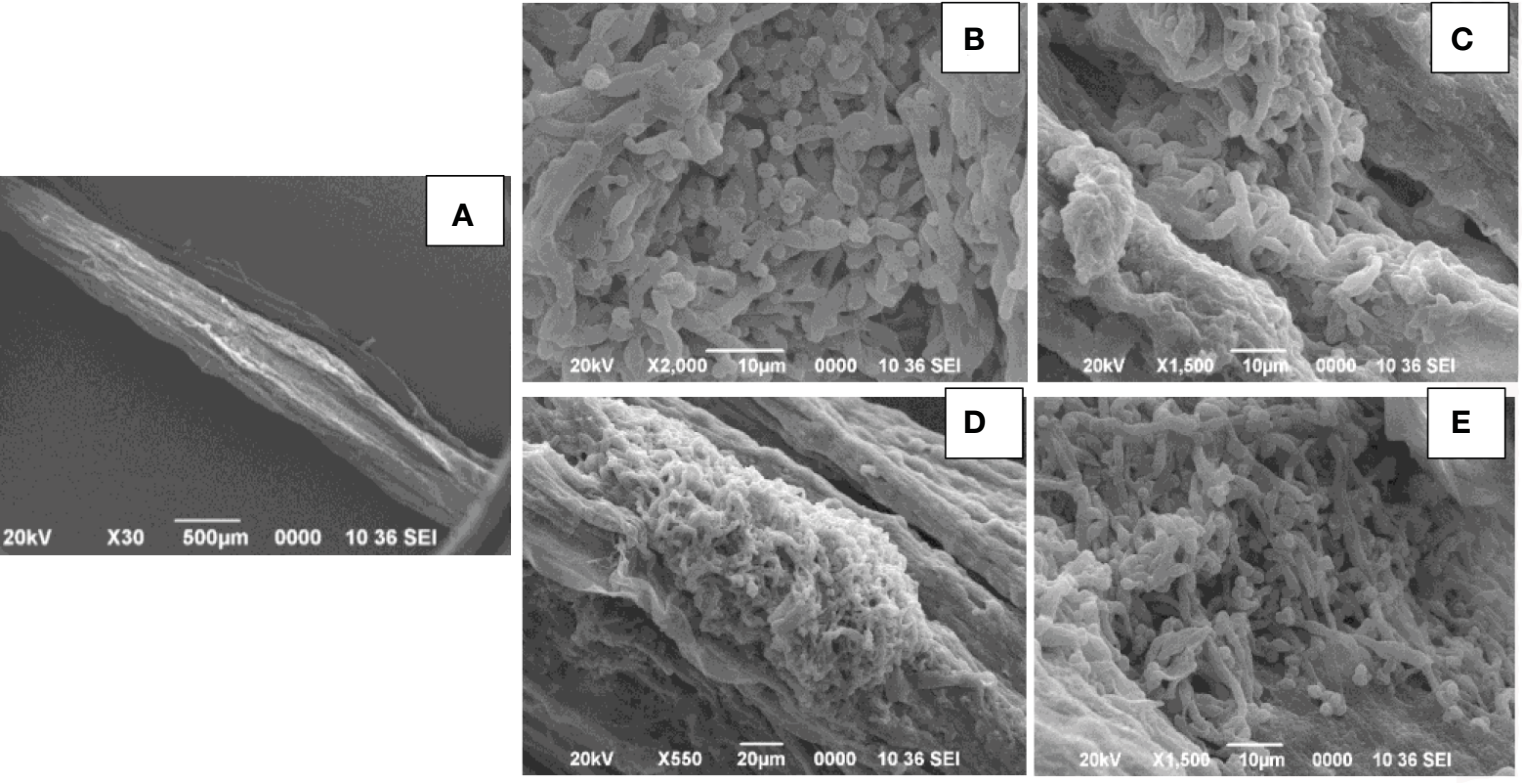
Scanning electron microscopy of primary roots of tomato seedling. **(A)**. Control: *Ralstonia solanacearum* inoculated- damaged roots. **(B–E)**. *Trichoderma harzianum* MC2 + *Trichoderma harzianum* NBG inoculated colonization of *Trichoderma* spp. in the root surface.

Seedlings treated with both *R. solanacearum* and *Trichoderma* were observed to be healthy when compared to wilted seedlings only treated with *R. solanacearum.* Primary root sections of the healthy tomato seedlings when examined by SEM revealed that mycelia of the isolates *T. harzianum* MC2 and *T. harzianum* NBG consistently colonized on the surface of roots (**Figure 12**). However, primary root sections of the untreated wilted plants revealed a damaged epidermal root surface.

## 4 Discussion

Dual culture antagonism assays reveal a significant reduction (p<0.05) in the mycelial growth of three fungal pathogens. This was likely due to the competition for available nutrients and space in the growing media (Anke, 1995; Chamedjeu et al., 2019). Three well-known mechanisms are associated with *Trichoderma* in regulating fungal pathogen growth: competition for nutrients, antibiosis, and myco-parasitism (Harman, 2006). Subtle differences are also observed in mycelial inhibition. This may be attributed to the mycelial diversity prevalent in *Trichoderma* stains (Anupama et al., 2015). In the present study, significant inhibition by *Trichoderma* was also ascertained in dual culture with *R. solanacearum*, supporting the use of the isolates in the management of bacterial wilt diseases. Such antagonism by both the fungal and bacterial pathogens may be attributed to the synthesis of certain molecules that inhibit the pathogen growth, as evident from the inhibition zone around its growth *in vitro* (Chamedjeu et al., 2019).

Additionally, *in vitro* assays were conducted to study the volatile and non-volatile antimicrobial compounds secreted by the bioagents against the pathogens. The isolates studied in the present work were evidently found to inhibit the mycelial growth of fungal pathogens within the range of 50-100%. The isolates studied in the present work were clearly able to inhibit 95-100% growth of the fungal pathogens. Such an antibiosis activity is possibly contributed by antifungal compounds released by the isolates which are evaluated subsequently in the present study. Active involvement of volatile and non-volatile organic compounds to inhibit fungal growth is previously reported (Morath et al., 2012). The emission of valuable volatile metabolites by *Trichoderma* species with antimicrobial properties inducing a defense response in plants is a notable discovery of recent times (Anupama et al., 2015; Wonglom et al., 2020). Most of the compounds identified belong to alcohols, acids, esters, ketones, and sesquiterpenes (Reino et al., 2008). Besides antagonism, the eight isolates stand positive for cellulase, amylase, and protease production test. The skeleton of pathogenic fungi cell walls contains chitin and enzymes, the enzyme capable of hydrolyzing these components must be released by a successful antagonist. A clear zone surrounding the microbial colonies is seen in the extracellular enzyme production. The qualitative assessment thus shows the ability to hydrolyze these enzymes, signifying their contribution as potent biocontrol agents. It is known that *Trichoderma* directly attacks the plant pathogen by excreting lytic enzymes by partial degradation of the pathogen cell wall (Haran et al., 1996).

The effect of two isolates, *T. harzianum* MC2 and *T. harzianum* NBG that stood out in the inhibition of mycelial growth of plant pathogens and biochemical assays were subsequently considered for biochemical evaluation of the antibiosis mechanism. It is a clear revelation from the LCMS analysis that the two best-performing isolates NBG and MC2 release a set of secondary metabolites previously recognized with anti-microbial activity. Apart from a few similar compounds, both isolates secrete different sets of metabolic compounds. As a result, despite belonging to the same taxon, the fungi may have different antibiosis mechanisms. A few compounds were predicted, while a few peaks from the LCMS analysis remained unidentified to previously reported metabolites, which may be indicative of novel compounds that aid in the antibiosis activity of the isolates and are subject to further investigation. Trichodin A, viridiol, 5-hydroxyvertinolide, pyridoxatin, alternariol monomethyl ether, and isoechinulin are released by *T. harzianum* NBG, which possess antifungal and antibacterial properties against different phytopathogens. Additionally, gliotoxin, harzianic acid, cremenolide, and cyclopenol are metabolites released by *T. harzianum* MC2 which are suspected to be antifungal in nature. The metabolomics analysis is evident and suffices for the biocontrol activity of the two agents.

Preliminary investigations on the diversity of the *Trichoderma* isolates performed by phylogenetic analysis provide significant taxonomic knowledge of the isolates. Among the eight isolates, most of them align distinctly with several other regions of India, based on the ITS phylogenetic marker. The isolate JC1 closely aligns with a *T. asperellum* (MW487267) isolated from groundnut rhizosphere. Similarly, the isolate NBY matches an endophytic *T. lixii* strain (KY425729) retrieved from the leaves of black rice. *T. harzianum* Thar12 (KU317844) isolated from soil from a vegetable field is phylogenetically similar to the isolates MC2 and JC1. Another isolate, *T. harzianum* KM14 (MK517774), which is reportedly derived from rhizospheric soil of ginger, is similar to the isolate NBG used in the current study. Because of the eminent genomic similarity, it may be inferred that the biocontrol activities established from the eight isolates may also harbor antibiosis activities if exploited against the aforementioned crops. However, NC1, NC2, and MCG are isolates that do not match any specific region of the Indian subcontinent. Such biodiversity may account for diverse applications in the field as biocontrol agents. Limited interspecific and intraspecific distances with no overlap help to separate the *Trichoderma* spp. in the current study. The isolates belong to the same species of genus Trichoderma, but show diverging evolutionary relationships in the phylogenetic tree, which can be further subjected to sequence polymorphism studies. It was found that the dataset obtained from the amplification of the *tef1α* region did not have a good number of similar matches with the isolates retrieved from NCBI. Furthermore, the analysis based on the maximum similarity percentage of the *tef1α* region of our isolates is exclusive of strains originating in the northeastern region, or any other part, of India.

We demonstrated the efficacy of the two strongest antagonistic bioagents (*T. harzianum* MC2 and *T. harzianum* NBG) in replicated field trials conducted during the 2021-2022 crop season of Rabi. The field data presented in the current study clearly establishes the potential of our formulation to work against bacterial wilt caused by *R. solanacearum* in solanaceous crops, which is a major disease of the Solanaceae family worldwide (Ahmed et al., 2022). Along with its efficacy in controlling wilt, the bioformulations in different forms of ingredients are also successful in promoting robust plant growth. The improved mechanism of biocontrol and growth enhancement may be attributed to the production of secondary metabolites and extracellular enzymes as seen in biochemical studies performed in this research work. Future research should investigate the potential impact of constitutive genes prominently implicated in mycoparasitism to the *in planta* efficacy.

## 5 Conclusion

Upon meticulous examination inclusive of *in vitro, in silico*, and *in planta* experiments, the conclusion of this study contributes to future research in the field of plant pathology as well as the emergence of potential biopesticide agents. In this regard, the use of the eight bioagents, in particular *T. harzianum* MC2 and *T. harzianum* NBG, for assessment within a disease management framework as eco-friendly and environment-friendly substitutes to pesticides is recommended. In addition, such a study integrated with a systems biology approach could further help to predict and characterize the microbiomes that will help to identify the underlying mechanisms for curbing pathogenic systems.

## Data availability statement

The datasets presented in this study can be found in online repositories. The names of the repository/repositories and accession number(s) can be found in the article/**Supplementary Material**.

## Author contributions

MR: conceptualization, methodology, investigation, writing- original draft preparation. SB: methodology, software, visualization, RT, RK: reviewing and editing. PB, ML: supervision, project administration. PKB: validation. BS: funding acquisition. All authors contributed to the article and approved the submitted version.
