# Supplementary material for: Deciphering the antimicrobial activity of multifaceted rhizospheric biocontrol agents of solanaceous crops viz., Trichoderma harzianum MC2, and Trichoderma harzianum NBG
Source: Front Plant Sci. 2023 Mar 3;14:1141506. doi: 10.3389/fpls.2023.1141506 (PMC10020943; doi:10.3389/fpls.2023.1141506)
Supplement: Supplementary file 1 [file DataSheet_1.docx]

Table S1. Location specific details of collected rhizospheric microbes

| **Agro climatic zone** | **Districts** | **Locations** | **Geographical coordinates** | |  | **Total no of soil samples collected and microflora isolated** | | | | | | |  |
| --- | --- | --- | --- | --- | --- | --- | --- | --- | --- | --- | --- | --- | --- |
|  |  |  |  |  | **Chili rhizosphere** | | | **Tomato rhizosphere** | | | **Brinjal colonies** | | |
|  |  |  | **Latitude (⁰N)** | **Longitude (⁰S)** | **SS^*^** | **BC^†^** | **FC^+^** | **SS** | **BC** | **FC** | **SS** | **BC** | **FC** |
| **Upper Brahmaputra Valley Zone** | Jorhat | Selenghat | 26.7526 | 94.4790 | 2 | 6 | 3 | 1 | 4 | 1 | 1 | 0 | 1 |
|  |  | Potiyagaon | 26.7934 | 94.2041 | 1 | 6 | 0 | 2 | 6 | 5 | 1 | 0 | 2 |
|  |  | Baligaon | 26.8340 | 94.1787 | 1 | 7 | 5 | 2 | 4 | 5 | 2 | 3 | 2 |
|  |  | **Total** | |  | **4** | **19** | **8** | **5** | **14** | **11** | **4** | **3** | **5** |
|  | Sivsagar | Demow | 27.1262 | 94.7428 | 3 | 5 | 2 | 3 | 7 | 4 | 1 | 0 | 0 |
|  |  | Nazira | 26.9098 | 94.7234 | 3 | 4 | 6 | 1 | 5 | 3 | 1 | 1 | 0 |
|  |  | Betbari | 27.0229 | 94.6827 | 3 | 6 | 0 | 1 | 4 | 1 | 1 | 2 | 0 |
|  |  | Geleki | 26.7959135 | 94.6915416 | 1 | 3 | 1 | 2 | 5 | 3 | 2 | 0 | 0 |
|  |  | **Total** | |  | **10** | **18** | **9** | **7** | **21** | **11** | **5** | **3** | **0** |
|  | Majula | Bhekulimari | 26.5702 | 94.1031 | 1 | 2 | 11 | 2 | 2 | 5 | 4 | 1 | 2 |
|  |  | Namkatoni | 26.5702 | 94.1033 | 7 | 11 | 5 | 6 | 5 | 13 | 3 | 0 | 1 |

|  |  | Lohali | 27.66544 | 95.57632 | 1 | 2 | 3 | 2 | 2 | 5 | 1 | 0 | 0 |
| --- | --- | --- | --- | --- | --- | --- | --- | --- | --- | --- | --- | --- | --- |
|  |  | **Total** | |  | **9** | **15** | **19** | **10** | **9** | **23** | **8** | **1** | **3** |
| **North Bank Plain Zone** | Darrang | Kharupetia | 26.5093 | 92.1374 | 2 | 4 | 3 | 3 | 2 | 6 | 1 | 2 | 4 |
|  |  | Dalgaon | 26.5534 | 92.2122 | 1 | 4 | 2 | 3 | 1 | 3 | 2 | 3 | 2 |
|  |  | **Total** | |  | **3** | **8** | **5** | **6** | **3** | **9** | **3** | **5** | **6** |
|  | Sonitpur | Napaam | 26.7070386 | 92.7778412 | 2 | 6 | 3 | 2 | 2 | 6 | 1 | 2 | 1 |
|  |  | Dhekiajuli | 26.7038397 | 92.4823707 | 4 | 10 | 17 | 3 | 6 | 8 | 1 | 3 | 2 |
|  |  | **Total** | |  | **6** | **16** | **20** | **5** | **8** | **14** | **2** | **5** | **3** |
| **Lower Brahmaputra Valley Zone** | Kamrup | Chhaygaon | 26.0481229 | 91.3868203 | 2 | 5 | 3 | 1 | 3 | 5 | 1 | 0 | 2 |
|  |  | Mirza | 26.2971276 | 91.6905328 | 2 | 7 | 2 | 3 | 4 | 3 | 2 | 3 | 5 |
|  |  | Hajo | 26.1908134 | 91.6468353 | 3 | 6 | 0 | 2 | 7 | 5 | 2 | 3 | 6 |
|  |  | **Total** | |  | **7** | **18** | **5** | **6** | **14** | **13** | **2** | **6** | **13** |
|  | Nalbari | Bangaon | 26.5792365 | 91.5426105 | 3 | 8 | 4 | 3 | 7 | 4 | 2 | 4 | 3 |
|  |  | Chamata | 26.4056198 | 91.3363786 | 1 | 4 | 2 | 2 | 3 | 6 | 1 | 3 | 5 |
|  |  | Borbhag | 26.5336356 | 91.5318392 | 4 | 11 | 5 | 1 | 2 | 4 | 1 | 6 | 4 |
|  |  | Ghograpar | 26.63424 | 91.24423 | 2 | 6 | 0 | 2 | 0 | 3 | 3 | 5 | 3 |
|  |  | **Total** | |  | **10** | **29** | **11** | **8** | **12** | **17** | **7** | **18** | **18** |

|  | Barpeta | Mandia, | 26.2758831 | 90.9761583 | 2 | 6 | 2 | 2 | 6 | 5 | 1 | 4 | 2 |
| --- | --- | --- | --- | --- | --- | --- | --- | --- | --- | --- | --- | --- | --- |
|  |  | Bhawanipur | 26.3301106 | 91.0322765 | 2 | 7 | 2 | 1 | 2 | 5 | 2 | 2 | 4 |
|  |  | Sorupeta | 26.317129 | 91.005405 | 3 | 8 | 7 | 2 | 6 | 4 | 1 | 3 | 5 |
|  |  | Sorbhog | 26.4893249 | 90.883607 | 5 | 16 | 12 | 2 | 3 | 6 | 1 | 3 | 2 |
|  |  | **Total** | |  | **12** | **37** | **23** | **7** | **17** | **20** | **5** | **15** | **13** |

**^*^SS: Soil Sample**

**^†^BC: Bacterial Colonies**

**^+^FC: Fungal Colonies**

Table S2. Micro-flora isolated and their inhibition percentages against four pathogens.

| **Sl no.** | **Isolates** | **Crop** | **Location** |  | **Inhibition percentages (%)** | |  |
| --- | --- | --- | --- | --- | --- | --- | --- |
|  |  |  | ***F. oxysporum* f. sp. *lycopersicum*** | | ***Colletotrichum gloeosporioides*** | ***Rhizoctonia solani*** | ***Ralstonia solanacearum*** |
| 1 | MB6 | Brinjal | Majuli | 65.88 | 77.77 | 50.33 | 19.00 |
| 2 | JB5 | Brinjal | Jorhat | 72.22 | 77.77 | 53.11 | 25.00 |
| 3 | MT10 | Tomato | Majuli | 71.11 | 78.11 | 55.55 | 21.00 |
| 4 | MB4 | Brinjal | Majuli | 74.44 | 66.66 | 58.33 | 26.00 |
| 5 | NT1 | Tomato | Nagaon | 65.33 | 66.44 | 59.77 | 23.66 |
| 6 | NT5 | Tomato | Nagaon | 73.11 | 67.00 | 62.22 | 20.66 |
| 7 | NT8 | Tomato | Nagaon | 51.11 | 78.66 | 67.00 | 18.66 |
| 8 | JT3 | Tomato | Jorhat | 78.11 | 78.66 | 69.77 | 30.00 |
| 9 | MT9 | Tomato | Majuli | 58.11 | 55.33 | 70.00 | 19.66 |
| 10 | BB1 | Brinjal | Barpeta | 62.22 | 77.77 | 71.11 | 16.66 |
| 11 | JT2 | Tomato | Jorhat | 66.60 | 77.77 | 72.22 | 35.17 |
| 12 | MT7 | Tomato | Majuli | 60.88 | 67.22 | 72.22 | 24.33 |
| 13 | BB2 | Brinjal | Barpeta | 58.88 | 89.44 | 74.44 | 14.66 |
| 14 | JC2 | Chili | Jorhat | 77.77 | 80.00 | 74.77 | 51.00 |
| 15 | NgC1 | Chili | Nagaon | 78.71 | 82.22 | 75.11 | 26.00 |
| 16 | NgC2 | Chili | Nagaon | 80.00 | 80.66 | 78.11 | 24.66 |
| 17 | MT4 | Tomato | Majuli | 53.33 | 67.00 | 78.33 | 24.00 |
| 18 | **NC2** | **Chili** | **Nalbari** | **81.80** | **88.66** | **88.88** | **38.51** |

| 19 | **NBY** | **Chili** | **Nalbari** | **81.80** | **88.66** | **91.11** | **34.81** |
| --- | --- | --- | --- | --- | --- | --- | --- |
| 20 | **MCG** | **Chili** | **Majuli** | **84.77** | **93.30** | **93.33** | **51.00** |
| 21 | **JC3** | **Chili** | **Jorhat** | **83.60** | **89.44** | **94.44** | **38.66** |
| 22 | **JC1** | **Chili** | **Jorhat** | **88.88** | **88.66** | **97.00** | **30.00** |
| 23 | **NC1** | **Chili** | **Nalbari** | **83.30** | **86.66** | **98.66** | **35.17** |
| 24 | **MC2** | **Chili** | **Majuli** | **92.50** | **91.44** | **100.00** | **38.33** |
| 25 | **NBG** | **Chili** | **Nalbari** | **88.80** | **94.44** | **100.00** | **46.29** |


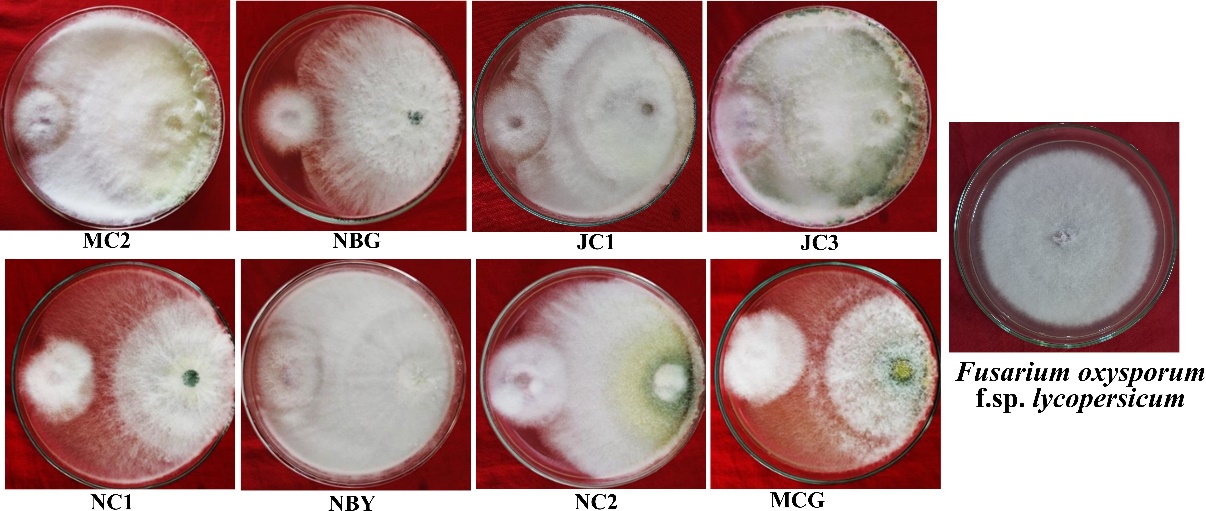


**Figure S1. Antagonistic activity of eight isolates against *Fusarium oxysporum* f. sp.**

***lycopersicum***


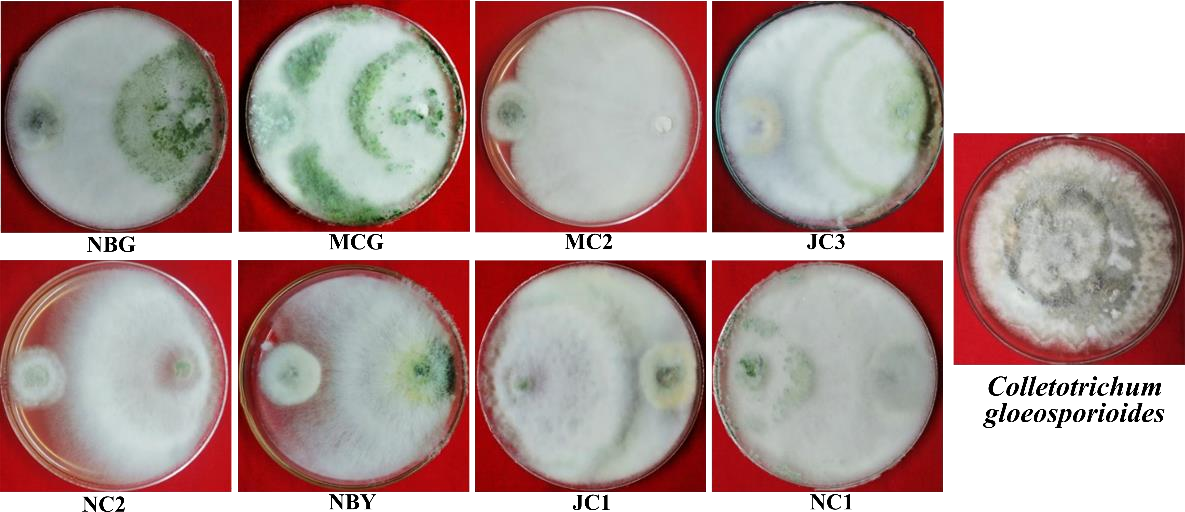


**Figure S2. Antagonistic activity of eight isolates against *Colletotrichum gloeosporioides***


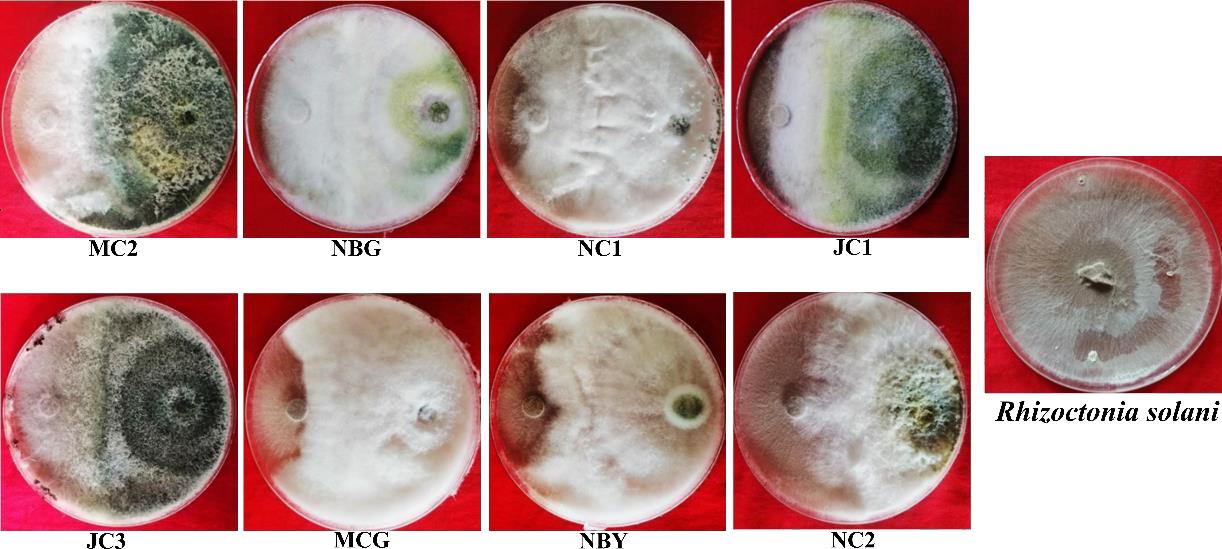


**Figure S3. Antagonistic activity of eight isolates against *Rhizoctonia solani***


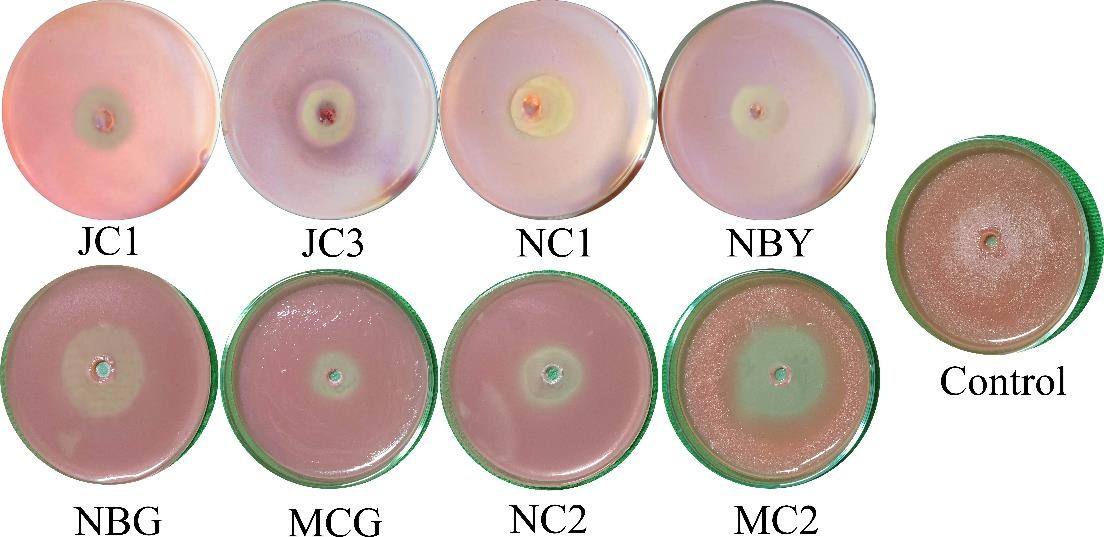


**Figure S4. Antagonistic activity of eight isolates against *R. solanacearum***


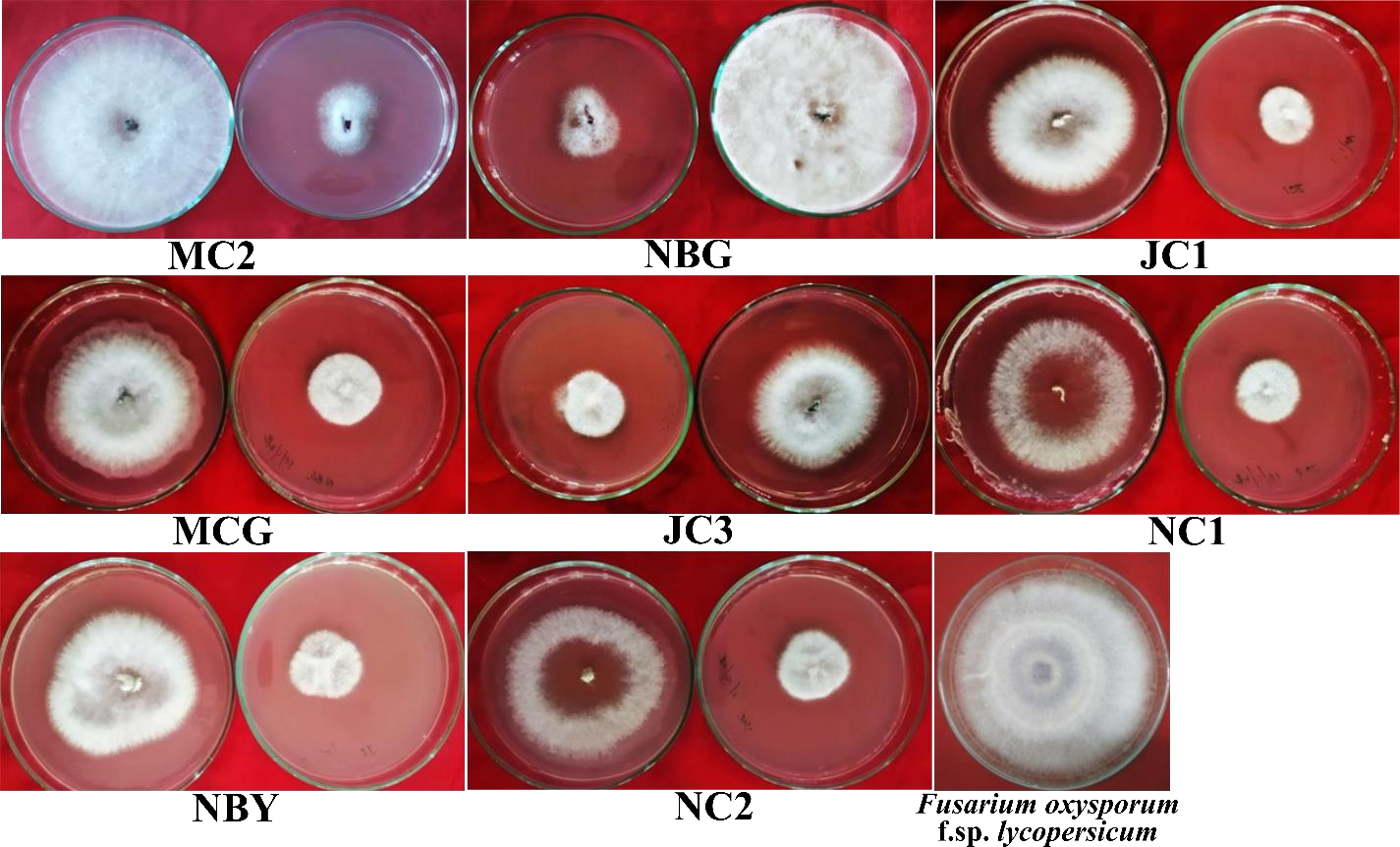


**Figure S5. Effect of volatile compounds produced by the eight rhizospheric isolates against *Fusarium oxysporum* fsp *lycopersicum*.**


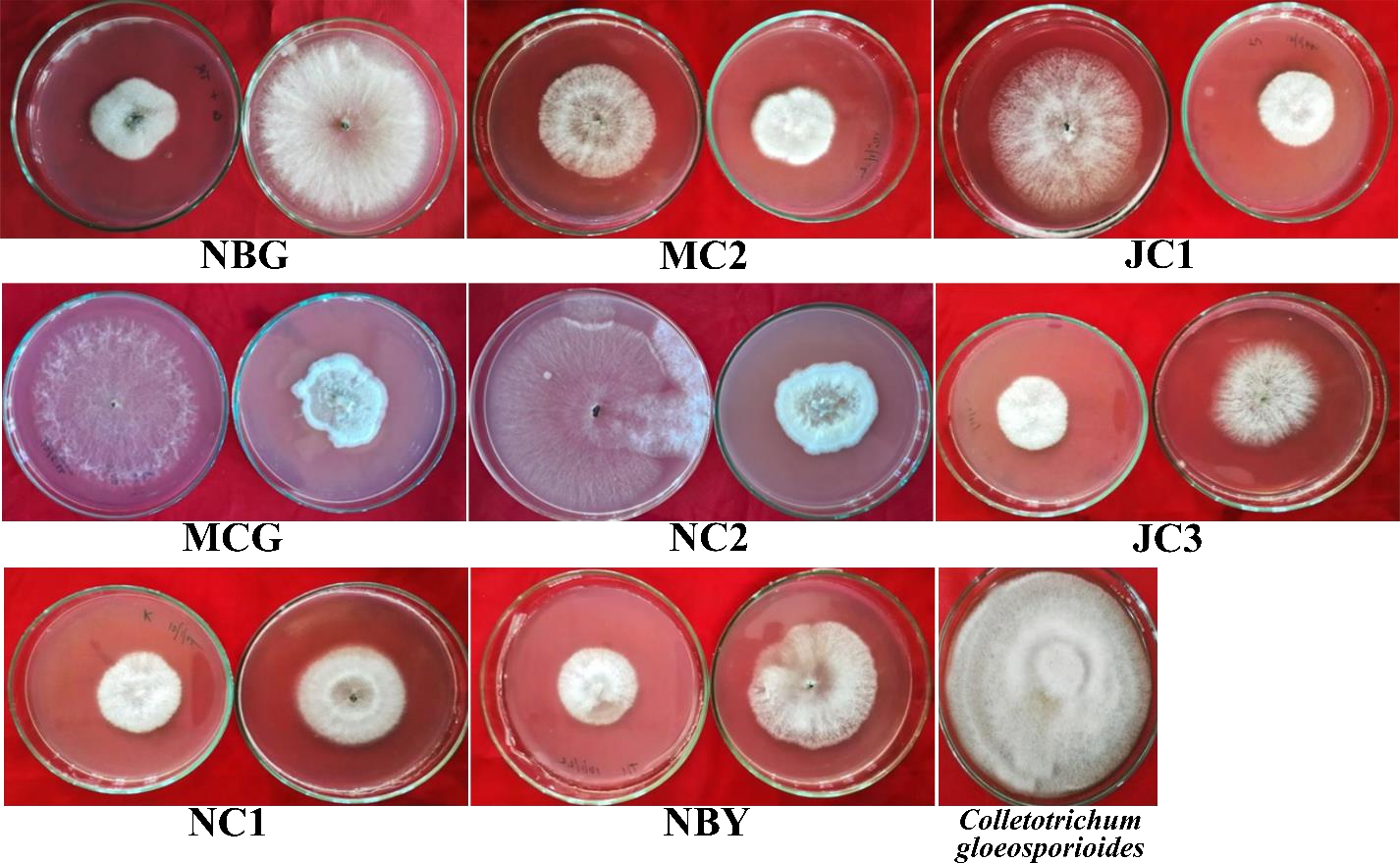


**Figure S6. Effect of volatile compounds produced by the eight rhizospheric isolates against *Colletotrichum gloeosporioides***


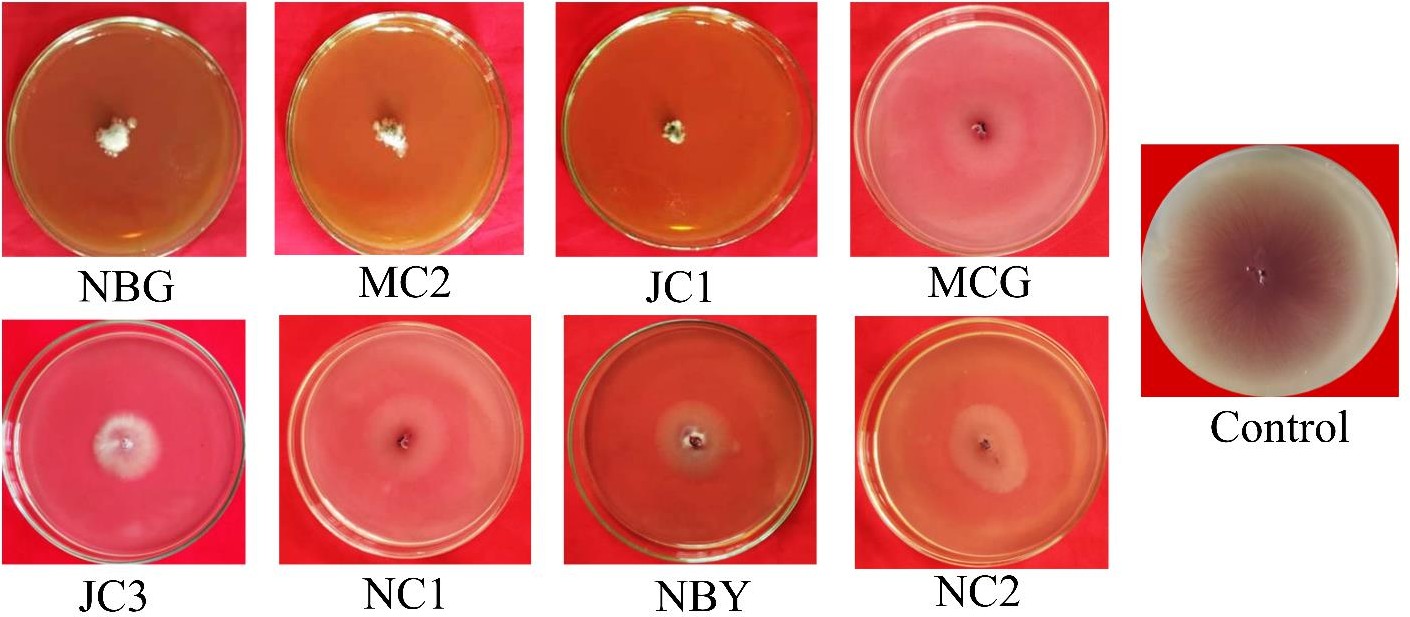


**Figure S7. Effect of non-volatile compounds produced by *Fusarium oxysporum* f. sp. *lycopersicum***


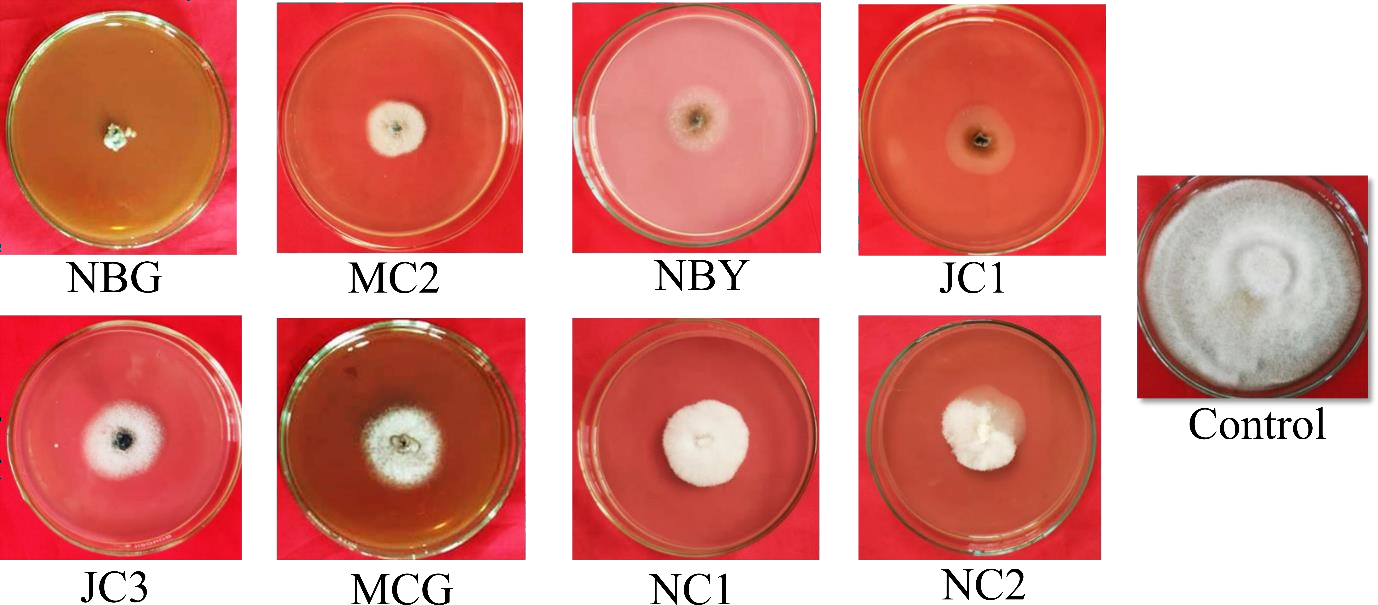


**Figure S8. Effect of non-volatile compounds produced by *Colletotrichum gloeosporioides***


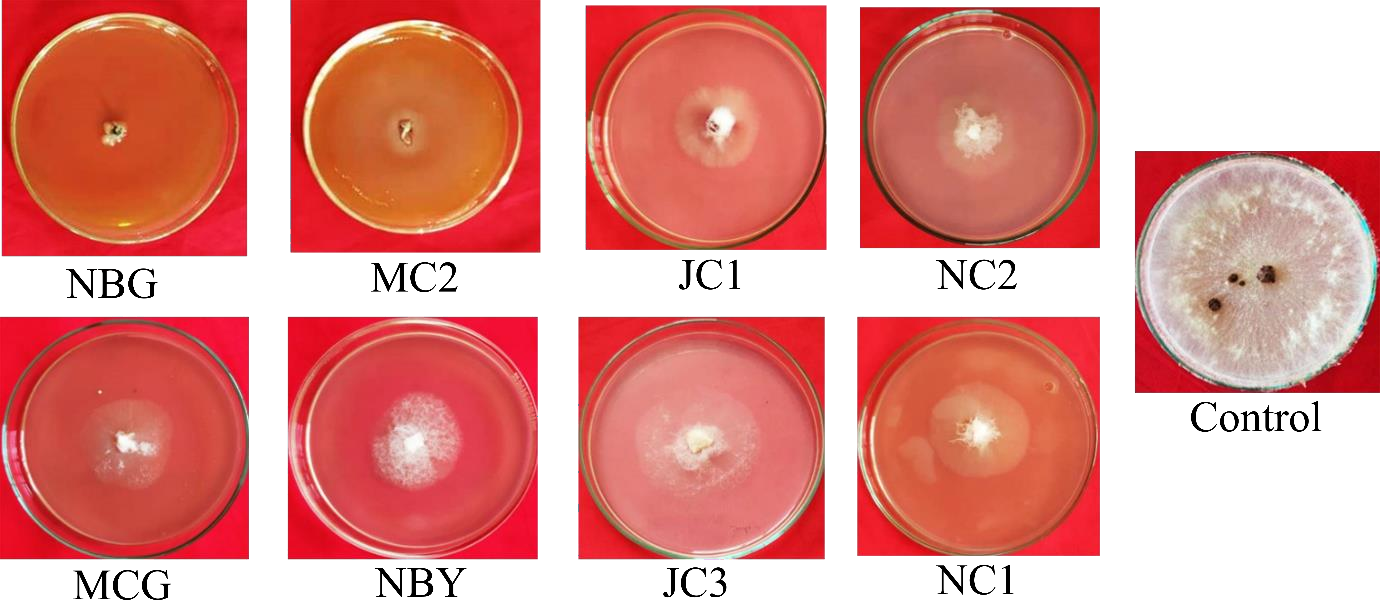


**Figure S9. Effect of non-volatile compounds produced by *Rhizoctonia solani***


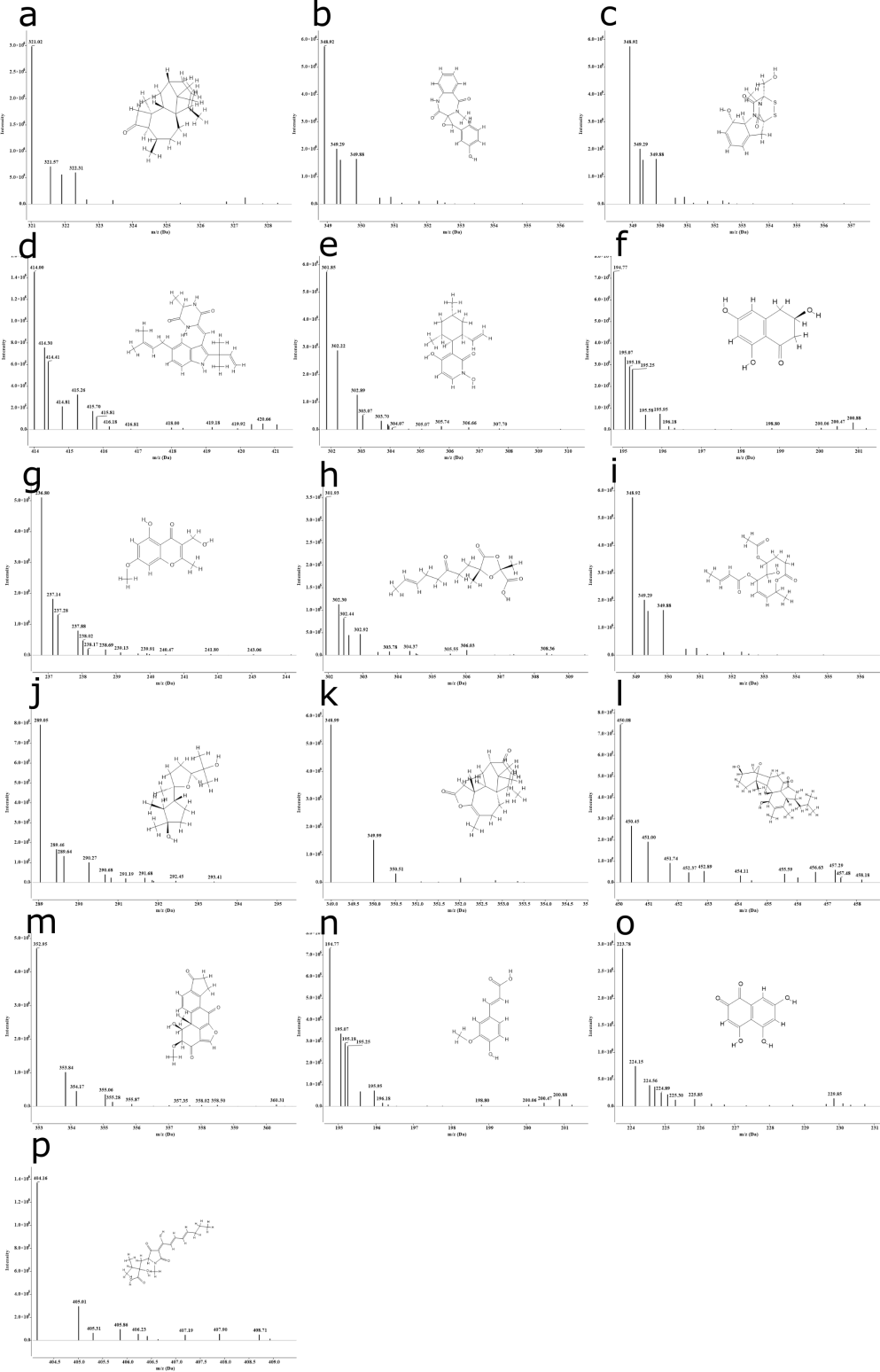


**Figure S10. Mass spectra of all compounds from LCMS extract of *Trichoderma harzianum* MC2.**

a. (9R,10R)-dihydro-harzianone, b. Cyclopenol, c. Gliotoxin, d. Isoechinulin A, e. Pyridoxatin, f. Scytalone, g. 5-Hydroxy-3-hydroxymethyl-2-methyl-7-methoxychromone,

h. Citrinoviric acid, i. Cremenolide, j. Cyclonerodiol oxide, k. Trichodermaerin, l. Trichoderone B, m. Viridin, n. Ferulic acid, o. Flaviolin, p. Harzianic acid.


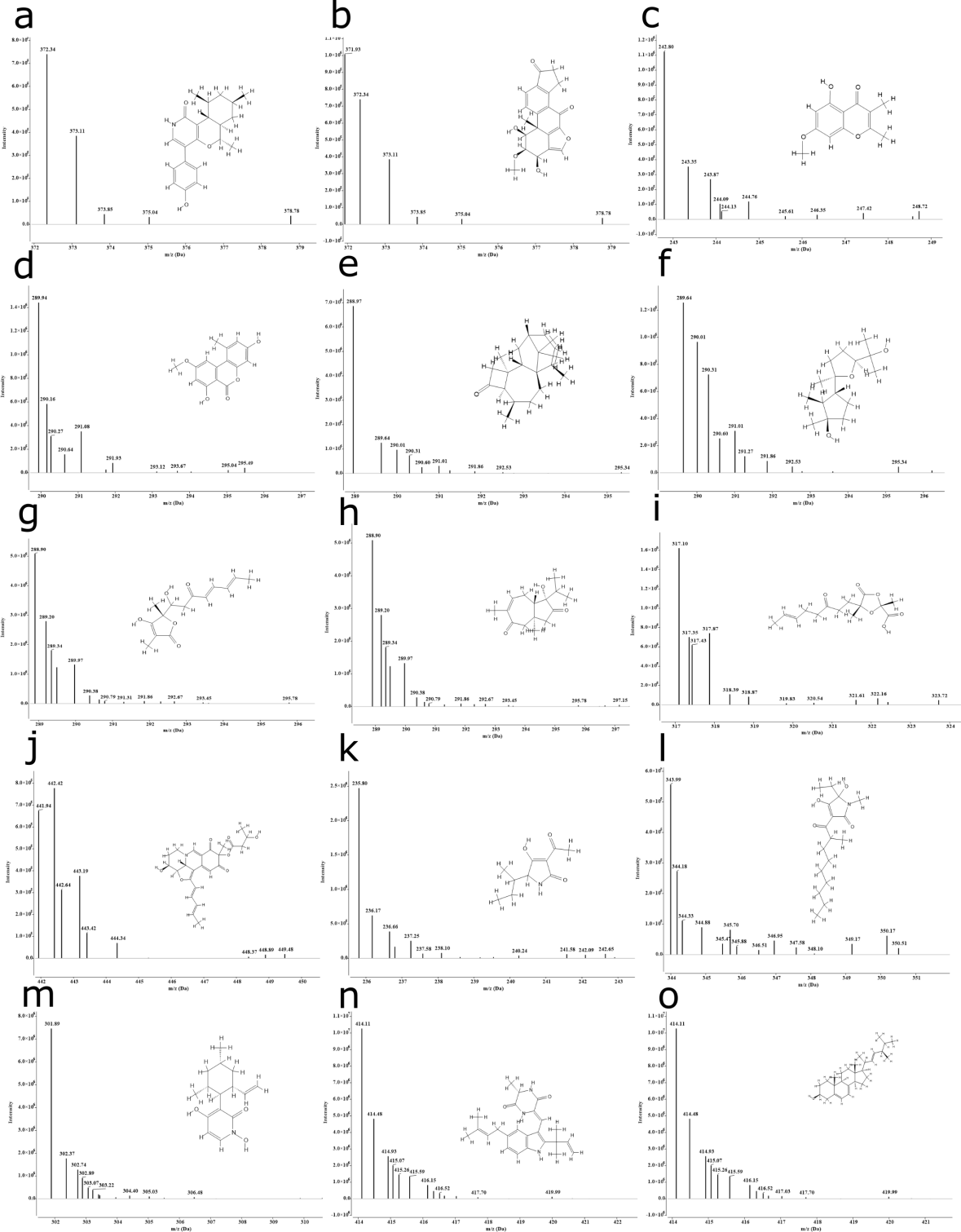


**Figure S11. Mass spectra of all compounds from LCMS extract of *Trichoderma harzianum* NBG.**

a. Trichodin A, b. Viridiol, c. 5-Hydroxy-3-hydroxymethyl-2-methyl-7- methoxychromone, d. Alternariol monomethyl ether, e. (9R,10R)-dihydro-harzianone, f. Cyclonerodiol oxide, g. 5-hydroxyvertinolide, h. Trichocarotin C, i. Citrinoviric acid, j. Fleephilone, k. Tenuazonic acid, l. Coprogen, m. Pyridoxatin, n. Isoechinulin A, o. Ergosterol
